# The genome of the glasshouse plant noble rhubarb (*Rheum nobile*) provides a window into alpine adaptation

**DOI:** 10.1038/s42003-023-05044-1

**Published:** 2023-07-10

**Authors:** Tao Feng, Boas Pucker, Tianhui Kuang, Bo Song, Ya Yang, Nan Lin, Huajie Zhang, Michael J. Moore, Samuel F. Brockington, Qingfeng Wang, Tao Deng, Hengchang Wang, Hang Sun

**Affiliations:** 1 CAS Key Laboratory of Plant Germplasm Enhancement and Specialty Agriculture, Wuhan Botanical Garden, Chinese Academy of Sciences, Wuhan, 430074 China; 2 CAS Key Laboratory for Plant Biodiversity and Biogeography of East Asia, Kunming Institute of Botany, Chinese Academy of Sciences, Kunming, Yunnan 650201 China; 3 Center of Conservation Biology, Core Botanical Gardens, Chinese Academy of Sciences, Wuhan, Hubei 430074 China; 4 Department of Plant Sciences, University of Cambridge, Tennis Court Road, Cambridge, CB2 3EA UK; 5 CeBiTec & Faculty of Biology, Bielefeld University, Universitaetsstrasse, Bielefeld, 33615 Germany; 6 Institute of Plant Biology & BRICS, TU Braunschweig, 38106 Braunschweig, Germany; 7 Department of Plant and Microbial Biology, University of Minnesota, Twin Cities, St. Paul, MN 55108 USA; 8 Department of Biology, Oberlin College, Oberlin, OH 44074 USA

**Keywords:** Plant evolution, Phylogenomics

## Abstract

Glasshouse plants are species that trap warmth via specialized morphology and physiology, mimicking a human glasshouse. In the Himalayan alpine region, the highly specialized glasshouse morphology has independently evolved in distinct lineages to adapt to intensive UV radiation and low temperature. Here we demonstrate that the glasshouse structure – specialized cauline leaves – is highly effective in absorbing UV light but transmitting visible and infrared light, creating an optimal microclimate for the development of reproductive organs. We reveal that this glasshouse syndrome has evolved at least three times independently in the rhubarb genus *Rheum*. We report the genome sequence of the flagship glasshouse plant *Rheum nobile* and identify key genetic network modules in association with the morphological transition to specialized glasshouse leaves, including active secondary cell wall biogenesis, upregulated cuticular cutin biosynthesis, and suppression of photosynthesis and terpenoid biosynthesis. The distinct cell wall organization and cuticle development might be important for the specialized optical property of glasshouse leaves. We also find that the expansion of LTRs has likely played an important role in noble rhubarb adaptation to high elevation environments. Our study will enable additional comparative analyses to identify the genetic basis underlying the convergent occurrence of glasshouse syndrome.

## Abstract (web summary)

A high-quality genome assembly and phylogenetic analysis for the glasshouse plant, *Rheum nobile*, shed light on its evolutionary history and ability to adapt to intensive UV radiation in a low-temperature, alpine environment.

## Introduction

Tertiary and Quaternary uplift of mountains has exposed organisms to demanding alpine conditions and accelerated the evolution of alpine biotas^1,2^. Plants have responded to harsh alpine environments with a high degree of specialization^1,3^, leading to diverse life forms including cushion plants, giant rosettes and succulents^3^. In Himalayan regions, the world’s most species-rich temperate alpine zone, specialized morphologies have evolved in response to hostile environmental conditions including low temperature, high solar radiation, strong winds and a short growing season^1^. Specialized morphologies include woolly plants (plants covered by dense woolly hairs), nodding plants (plants with flowers facing the ground) and glasshouse plants. Glasshouse plants are perhaps the most striking, with inflorescences sheltered by semi-translucent leaves that create a warmer interior and can be compared to the glass in a greenhouse^4–6^. The most prominent Himalayan alpine plant is noble rhubarb [*Rheum nobile* Hook.f. & Thomson, Polygonaceae)], which is the flagship glasshouse plant adapted to high elevations (>4000 m)^7^.

In contrast to sympatric competitors that generally are dwarf or prostrate, noble rhubarb grows to heights of up to 2 m when in flowering, making it highly conspicuous in the alpine region. The remarkable glasshouse-like morphology was assumed to be essential for the plants to cope with low temperature and strong UV radiation at high elevation^8–10^. This adaptive trait has also been found to be important for the mutualism between the plants and the pollinating seed-consuming *Bradysia* fungus gnats, providing shelter for adult oviposition and larva development^11–13^.

Elucidating the genetic basis of adaptive traits is a central goal of evolutionary genetics^14^. Genomic modification associated with adaptation to high elevations has been well documented in animals^15^ but has been less explored in plants relative to the high diversity of alpine flora. But as iconic plants of the alpine landscape, glasshouse plants have become the focus of greater ecological and evolutionary interests^8,10,12,13,16–19^. Studies on the ecological function of the specialized structures of alpine plants, such as cushion-like leaf canopy^20–23^, hairy leaves and inflorescences^24–26^, leafy bracts^8,12,13^, and nodding capitula^27,28^ have revealed that these traits are particularly efficient in heat-trapping. For example, the temperature within the leaf canopy of the cushion plant *Silene acaulis* (L.) Jacq. is typically 15 °C higher than the ambient temperature during clear summer days^20^. Such thermal benefits are essential for growth, development, metabolism and reproduction of plants that inhabit the consistently cold, windy environments of alpine regions^25^. In addition, the downward orientation of flowers and glasshouse-like leaves are assumed to be helpful in protecting the sensitive reproductive parts from UV radiation and frequent storms^10,12,13,27–29^. Despite great progress toward understanding the functional ecology of these extremophiles, the genetic basis facilitating their fascinating adaptation is poorly understood, owing to the lack of genomic information.

Here, we report the genome sequence of the flagship glasshouse plant noble rhubarb and integrate comparative genomic, transcriptomic, and phytochemical data to provide insights into the evolution of glasshouse morphology. We show that the glasshouse leaves function as solar radiation filters that absorb UV light to provide photoprotection for reproductive organs, and reveal that this adaptive morphological syndrome has cryptically evolved in at least three lineages in rhubarb. By deciphering the transition pattern of the glasshouse syndrome, both in morphology and in transcriptomic profiles, we identified key genetic network modules underlying the developmental differentiation of glasshouse leaves from normal leaves. The data presented in this study lay the foundation for further deciphering the genetic mechanisms underlying the origin and evolution of glasshouse morphology.

## Results

### Multiple origins of the glasshouse syndrome in *Rheum* L.

**Fig. 1:**
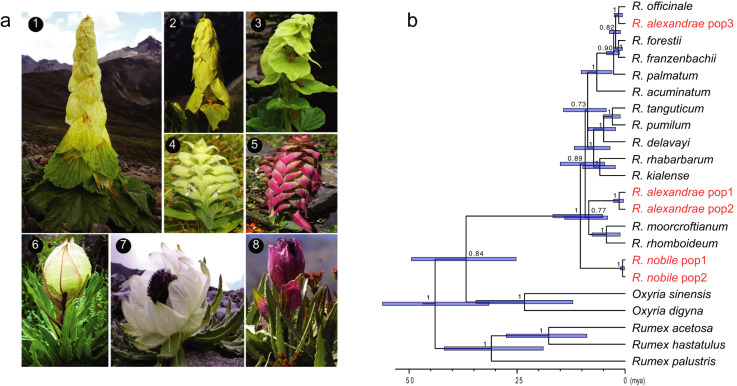
Glasshouse plant morphology and its evolution in *Rheum*. **a** Representatives of glasshouse plants. A–C: *Rheum nobile*, *R. alexandrae var*. 1 and *R. alexandrae var*. 2 (Polygonaceae). D, E: *Ajuga lupulina* var. *lupulina* and *A. lupulina* var. *major* (Lamiaceae). F–G: *Saussurea obvallata*, *S. involucrata* and *S. velutina* (Asteraceae). **b** Phylogeny of *Rheum* estimated from 132 orthologs using coalescent method with local posterior probabilities shown in node. The glasshouse lineages are labeled in red. Node bars represent 95% highest posterior densities (HPD) of divergence time estimated using MCMCTree.

Glasshouse plants are unique in the Eastern Asian alpine biome and are recorded in several phylogenetically distant plant taxa, including the flowering plant families Lamiaceae, Asteraceae, and Polygonaceae (Fig. 1a). The most notable glasshouse-like morphology is found in *Rheum* L. (Polygonaceae), such as noble rhubarb, a wild relative of commercial rhubarb. Noble rhubarb is commonly named yellow tower in Chinese because the yellowish leaves are reflexed and form a compact tower-like structure covering the sensitive reproductive organs (Fig. 1a). Of the 60 species described in *Rheum*, two glasshouse plant species, *R. nobile* and *R. alexandrae*, have been recorded^7^. To track the origin and evolution of the glasshouse syndrome in *Rheum*, six newly generated transcriptomes together with 16 publicly available transcriptomes covering four of the six sections of *Rheum* were used in phylogenomic reconstruction (Supplementary Data 1). Previous phylogenetic analyses^30^ using a few cpDNA fragments failed to resolve the deep phylogenetic relations within *Rheum*. Our phylogenomic analysis with 132 genes generated a high-quality phylogeny of *Rheum* (Fig. 1b). *R. nobile* was sister to the rest of *Rheum* sampled, while the accessions of *R. alexandrae* were polyphyletic and recovered in two different clades that contain non-glasshouse species (Fig. 1b). *R. alexandrae* is widely distributed in the Hengduan mountain region, and only a single accession has been included in previous molecular phylogenies^30,31^. Given the remarkable morphological divergence (Fig. 1a: B, C), and the polyphylogeny of *R. alexandrae* revealed here, a third, previously unrecognized origin of glasshouse syndrome is plausible. Further analysis including more populations and closely related species is needed to clarify the circumscription of *R. alexandrae*. In addition, the densitree of *Rheum* generated from 187 single-copy genes showed reticulated relationships among some lineages (Supplementary Fig. 1). Nonetheless, at least three independent origins of the glasshouse syndrome in *Rheum* are inferred based on the sampling and phylogeny presented here.

### The noble rhubarb glasshouse is highly effective in blocking UV but transmits visible and infrared light

**Fig. 2:**
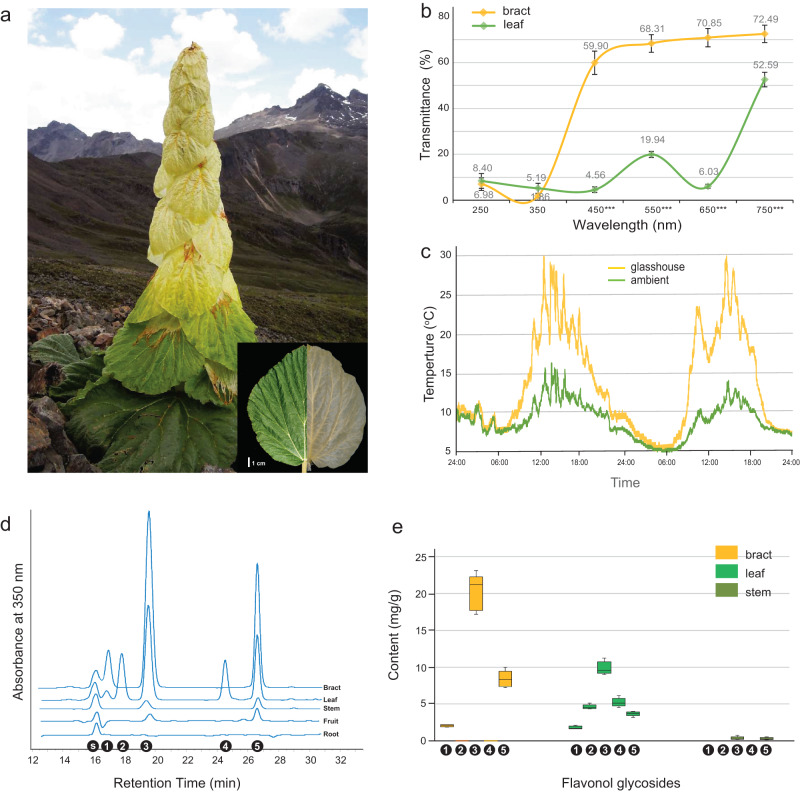
The translucent leaves in the noble rhubarb function as glasshouse. **a** A mature noble rhubarb plant in the Hengduan Mountains (N28.53°, E99.95°; Elev. 4500 m) with insert showing a morphological comparison between a photosynthetic basal leaf and a translucent leaf. **b** Spectral characteristics of bracts and leaves in light transmittance, showing the different patterns of light transmittance by bracts and leaves. Significance was statistically tested at six wavelengths (250–750 nm) using *T* test (*n* = 3 biologically independent samples; ***p < 0.001). **c** Temperature change inside and outside of the inflorescence glasshouse in a 48 h interval at mid-August in the eastern Himalayas (N29.40°, E94.90°; Elev. 4200 m). **d** The HPLC spectrum of UV-absorbing substances extracted from five tissue types; s: the flavonol standard isovitexin; 1–5: the compounds isolated from tissues. **e** The contents of the five flavonol species isolated from noble rhubarb tissues. Fruits and roots only contained traces of the flavonols and are not shown. In boxplots, the center, lower, and upper lines depict the median, 25th, and 75th percentile respectively, and whiskers represent maxima and minima.

The glasshouse structure is composed of specialized cauline leaves which are termed as bracts (used to represent the yellowish glasshouse leaves hereafter) (Fig. 2a). By directly measuring the reflectance and transmittance spectra of fresh tissues in the wild using a spectrophotometer equipped with an integrating sphere (Supplementary Fig. 2), we demonstrated that the semi-translucent bracts of noble rhubarb can block >95% UV radiation (250–400 nm) while transmitting 60–80% visible and infrared light (>400 nm) (Fig. 2b, Supplementary Figs. 3, 4). In contrast, the green leaves effectively block both UV light and most visible light (Fig. 2b, Supplementary Fig. 4). *T*-test show that the light transmission rate in visible range (400–750 nm) is significantly higher in bracts than in leaves (*p* < 0.01). Our results revealed that the bract is more effective as a light filter than previously assumed^8,10^, probably because we used the fresh tissue for measurements and we used scattered light that more closely resembles natural conditions. However, our limited sampling limits any insight into variation among individual plants. Further measurements in population level are needed to explicitly characterize the natural variation in glasshouse morphology. In addition, both bracts and leaves reflected a negligible proportion of UV radiation (Supplementary Fig. 4), indicating that the UV light was largely absorbed by the tissue rather than being reflected. UV radiation is intense at high elevations and can cause pollen malformation and reproductive sterility^32^. It is plausible that this spectral characteristic of the bracts may generate a favorable microclimate for the reproductive organs to develop.

In addition to UV radiation, we further evaluated this microclimate hypothesis by measuring changes of temperature in the ambient environment versus inside the leafy glasshouse at a 48 h interval during the growth season in the field. During the daytime (08:00–20:00), the internal temperature was constantly higher than the external temperature and reached as high as 25 °C around noon, while the ambient temperature was lower than 15 °C at the same time (Fig. 2c). An optimal flower temperature has been shown to be crucial for plant reproduction^33^, as temperature mediates flower development, pollen viability and pollen tube growth, and influences pollinating insect activity. Our previous control experiments found that the reproductive fitness of noble rhubarb significantly decreased when the bracts were removed^13^. These observations indicate that the glasshouse morphology is an important adaptive trait for alpine environments.

It has been widely acknowledged that flavonoids can function as photoprotection molecules for land plants because of their high efficiency in scavenging UV-induced reactive oxygen species. To explore how flavonoids function in noble rhubarb in resistance to UV radiation, we examined flavonoid content in five different organs (bract, leaf, stem, fruit and root). Using high performance liquid chromatography (HPLC), we isolated five UV-absorbing compounds from noble rhubarb organs (Fig. 2d, Supplementary Fig. 5). On the basis of the mass spectra and co-chromatography with an authentic standard, the compound was identified as quercetin 3-O-rutinoside (C1), quercetin 3-O-glucoside (C2), quercetin-3-O-galactoside (C3), quercetin 3-O-arabinopyranoside (C4,) and quercetin 3-O-[6´´- (3-hydroxy-3-methylglutaroyl)-glucoside] (C5) (Supplementary Figs. 6–10). All these UV-absorbing substances were quercetin-based glycosides, a subclade of flavonols that have been considered as photoprotective compounds for plants because of their UV-absorbing characteristics^34,35^. It is notable that, in addition to bracts, the leaves also accumulate large amounts of flavonols, while the other organs (stem, fruit and root) contain only traces of them (Fig. 2d, e). Leaves and bracts contain comparative levels of flavonoids as a whole, while bracts are specialized by losing quercetin 3-O-glucoside and quercetin 3-O-arabinopyranoside (Fig. 2d, e).

Following a phylogenetic approach, we identified putative noble rhubarb genes that encode the enzymes in the flavonoid biosynthesis pathway and characterized their expression profile using transcriptome data. Notably, chalcone synthase (*CHS*), which encodes the enzyme that directs phenylpropanoid metabolic flux to the flavonoid pathway, is expanded to 5 members (Supplementary Fig. 11). Some of these *CHS* copies display tissue-specific expression patterns. Consistent with our observation that quercetin-derived flavonols are the major UV-absorbing substances accumulated in bracts and leaves, at least one copy of the key gene flavonol synthase (*FLS*, Rn_tig2787.230) is highly expressed in bracts and leaves but is scarcely expressed elsewhere (Supplementary Fig. 11). In addition, the flavone synthase gene (*FNS*, Rn_tig2803.307) which competes with flavanone-3-hydroxylase (*F3H*) for the substrate to produce flavones, is not expressed in mature plants (Supplementary Fig. 11).

### Sequencing, assembly and annotation of the noble rhubarb genome

**Fig. 3:**
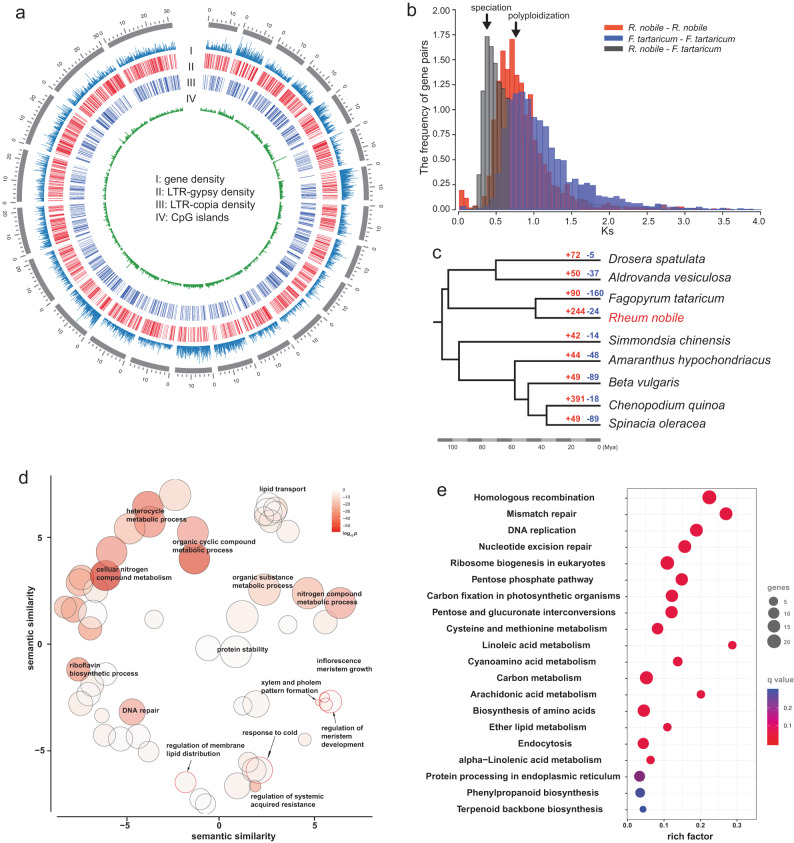
Genome architecture and gene family expansion and contraction. **a** Multi-dimensional display of genomic components of the noble rhubarb genome. The density was calculated per 100 Kb. **b** Frequency distributions of synonymous substitution rates (Ks) between homologous gene pairs in syntenic blocks of *R. nobile-R. nobile*, *F. tataricum-F. tataricum* and *R. nobile-F. tataricum*. Both species are members of the family Polygonaceae. **c** Number of significantly expanded (+) and contracted (-) gene families in nine Caryophyllales species with high-quality genome sequences available. **d** REVIGO clusters of overrepresented GO terms for significantly expanded gene families in the noble rhubarb genome. Each bubble represents a summarized GO term from the full GO list by reducing functional redundancies, and their closeness on the plot reflects their closeness in the GO graph, i.e. the semantic similarity. **e** KEGG enrichment of metabolic genes that were significantly expanded in the noble rhubarb genome.

We generated 116.35 Gb PacBio long reads (78× coverage) and 155.4 Gb (105× coverage) cleaned Illumina short reads for noble rhubarb (Supplementary Data 2). Long reads were used for de novo assembly, which was corrected and polished with short reads (the full assembly pipeline is shown in Supplementary Fig. 12), resulting in a 1.36 Gb draft genome sequence with a contig N50 of 9.8 Mb (Fig. 3a). The draft genome sequence comprises 245 contigs and accounts for 94% of the genome size of about 1.48 Gb as estimated by k-mer distribution with high frequency k-mer (>1k) excluded (Supplementary Fig. 13). BUSCO assessment recovered 94.5% complete genes from the assembly (BUSCO v5.1.2 with eudicotyledons_odb10 database, Supplementary Fig. 14), and the Merqury^36^ kmer plot (Supplementary Fig. 15) shows a main single peak which corresponds to a haploid assembly. These results attest to the completeness of the noble rhubarb genome sequence (Table 1, Supplementary Data 2). Using a combination of ab initio and hint-based methods (Supplementary Fig. 16), 58,950 high confidence gene models were predicted.

**Table 1:** Statistics for the genome assembly of noble rhubarb.

Assembly	
Assembled size	1.36 Gbp
GC content	39%
Number of contig	245
N50 of contig	9.8 Mbp
Longest contig	23.56 Mbp
BUSCOs	C:96.1% (S:87.3%, D:7.2%), F:1.6%, M:3.9%
Merqury QV & Error rate	46.3 & 2.33471e−05
Merqury k-mer completeness	99.24%
LAI (LTR Assembly Index)	9.90
Annotation	
Number of protein coding genes	58,950
Number of tRNA	3310
Number of sRNA	4798
Content of repeats	1,002.07 Mbp (73.54%)
-LTR	552.44 Mbp (40.54%)
-DNA transposons	102.44 Mbp (7.52%)
-LINEs	49.85 Mbp (3.66%)
-SINEs	1.53 Mbp (0.11%)
-Unclassised Repeats	279.86 Mbp (20.54%)

Ks plots of homologous colinear gene pairs revealed a main peak at 0.73 in *R. nobile* (Fig. 3b, Supplementary Fig. 17a). To characterize this potential genome polyploidization event, we further performed Ks calculations for *Fagopyrum tataricum* paralogous gene pairs and *R. nobile*-*F. tataricum* orthologous gene pairs. A similar Ks peak was also observed for *F. tataricum* paralogs and the mean Ks value for the *R. nobile*-*F. tataricum* orthologs is 0.48 (Fig. 3b). The divergence time for *R. nobile*-*F. tataricum* is around 45 mya^37^, thus the age of the polyploidization (Ks = 0.73) is 68.43 mya (T = Ks/2*μ*). Therefore, the polyploidy event is shared by both *R. nobile* and *F. tataricum. F. tataricum* and *R. nobile* are both from the buckwheat family Polygonaceae, which have an ancient genome polyploidization based on both Ks and gene tree approaches (Supplementary Fig. 17b). We then examined the syntenic depth of genic blocks between *R. nobile*/*F. tataricum* and beet (*Beta vulgaris*) that lacks genome polyploidization after the core-eudicots shared gamma event^38^. The syntenic pattern 1-1 (20%), 1-2 (23%), 1-3 (20%) or 1-4 (12%) was observed in *B.vulgaris*-*R.nobile* comparison, similar to the ratio in *B.vulgaris-F. tataricum* comparison (Supplementary Fig. 18). Thus, WGT and WGD cannot be distinguished based on this data. Nonetheless, these data indicated that both *R. nobile* and *F. tataricum* genomes have both undergone substantial genome fragmentation after the early Polygonaceae polyploidization event. This is further supported by the overall genome synteny between *B. vulgaris, R. nobile and F. tataricum* (Supplementary Fig. 19).

In context of the evolution of the Caryophyllales (Supplementary Data 3), we examined gene family expansion/contraction in noble rhubarb. We identified 244 gene families that are significantly expanded in noble rhubarb (Fig. 3c). We then performed Gene Ontology (GO) enrichment analysis to explore functions associated with these expanded gene families. The expanded gene families are enriched significantly in 46 GO terms including response to cold, DNA repair, and regulation of protein stability (Fig. 3d). Among these, several GO terms are of particular interest regarding alpine adaptation (Supplementary Data 4). The first is “response to cold” (GO: 0009409, *p* = 4.4e−9) which is self-explanatory. The second is “regulation of membrane lipid composition” (GO: 0010876, *p* = 3.6e−15). Other significantly overrepresented GO categories included inflorescence meristem growth (GO: 0010081, *p* = 5e−10; GO: 0099009, *p* = 3.1e−4), regulation of protein stability (GO: 0031647, *p* = 3.6e−2) and response to temperature stimulus (GO: 0009266, *p* = 1.8e−4). Kyoto Encyclopedia of Genes and Genomes (KEGG^39^) enrichment analyses revealed significant overrepresentation of 18 pathways (*Q* value < 0.20 after multiple test correction, Fig. 3e). The top four pathways enriched specifically in noble rhubarb are all related to DNA repair (Fig. 3e). Other enriched KEGG terms included linoleic acid metabolism, ether lipid metabolism, and alpha-linolenic acid metabolism (Fig. 3e), all of which are related to the biosynthesis of unsaturated fatty acids that can increase cell membrane fluidity in response to cold stress^40^.

### The noble rhubarb genome is rich in LTRs

**Fig. 4:**
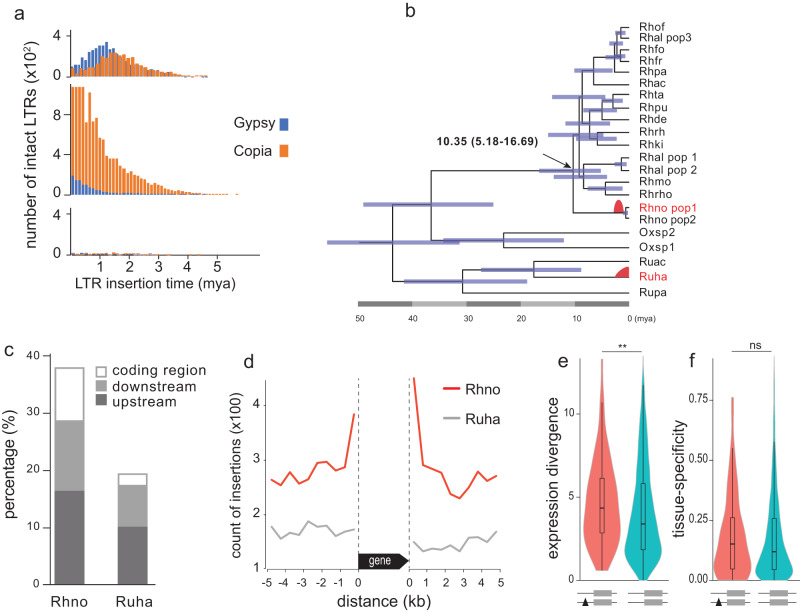
Evolutionary dynamics of LTRs. **a** Histogram of intact LTR insertions in *R. nobile* (top), *Ru. hastalutus* (middle) and *F. tataricum* (bottom). **b** Time-calibrated phylogeny of *Rheum* and its close relatives *Rumex* and *Oxyria* with the proliferation of LTRs labeled with red shapes. **c** Proportion of intact LTR insertions within the coding region of annotated genes and downstream (5 kb) or upstream (5 kb) of annotated genes in *R. nobile* (Rhno) and *Ru. hastalutus* (Ruha). **d** LTR insertions were enriched in the flanking regions of coding sequences (1 kb) in Rhno but not in Ruha. **e**, **f** Violin plots of the divergences (log_10_ transformed) between gene pairs at expression and tissue specificity levels (Wilcoxon rank sum test). Boxplots represent the median, 25th, and 75th percentile, respectively.

A much higher proportion of repetitive sequences was observed in noble rhubarb (77.46%) than in *F. tataricum* (Tartary buckwheat, 50.96%; Supplementary Fig. 20). Long terminal repeats (LTRs) are the most abundant elements and account for 42.91% of rhubarb genome. To investigate the evolutionary dynamics of LTRs in rhubarb, we re-assembled and annotated the genome of *Rumex hastatulus*^41^ which is in the sister genus to *Rheum*. The *Ru. hastatulus* genome has also significantly expanded compared to *F. tataricum*, and repetitive sequences make up 83.29%. Similarly, LTRs contribute most to the genome expansion, accounting for 63.05% of *Ru. hastatulus* genome. Following the same procedure, 10619 and 18863 intact LTRs were identified from *R. nobile* and *Ru. hastatulus*, respectively, while only 775 were detected in Tartary buckwheat (Fig. 4a). Analyzing the history of LTR insertions revealed distinct LTR evolutionary dynamics among them. LTRs have proliferated in *R. nobile* and *Ru. hastatulus* (Fig. 4a), however, the proliferations in *R. nobile* peaked at ~1-2 Mya, while proliferation in *Ru. hastatulus* was dominated by more recent LTR insertions within the past 0.8 Mya (Fig. 4a, b). In addition, Gypsy LTR is the main type in *R. nobile* while Copia LTR dominates in *Ru. hastatulus* (Fig. 4a).

Further inspection of LTR proliferation identified stronger association between LTRs and gene coding loci in *R. nobile* than in *Ru. hastatulus*, with 37.8% of the total intact LTRs found inserted near (<5 kb) the coding sequences in *R. nobile*, while the proportion was 19.5% in *Ru. hastatulus* (Fig. 4c). A closer look at the insertions indicated that the insertions were enriched at the flanking sequences of genes (<1 kb) in the *R. nobile* but not in *Ru. hastatulus* (Fig. 4d). The enrichments observed in glasshouse plants could be the results of insertion preference of TEs, as was observed in rice^42^.

To test this hypothesis, we then analyzed the solo LTRs, which are the remnants of intact LTRs by LTR removal mediated by interelement recombination. Large amounts of solo LTRs (>100 k) were identified in both species (Supplementary Fig. 21), indicating high frequency and efficiency of LTR removal in addition to active LTR amplification. These solo LTRs were distributed evenly on chromosomes in terms of their association with genes (Supplementary Fig. 22), suggesting that the insertion of LTRs was random, without preference to genic region in both species.

To test the functional consequences of LTR insertion on genome-wide gene expression profile, we focused on the gene pairs that are derived from recent gene duplications (Ks <0.5) (Supplementary Fig. 16) and analyzed the gene expression divergence and tissue specificity. The pairs that had LTR insertions in <1 kb flanking region had significantly higher levels of divergence in expression (Fig. 4e), although not in tissue specificity (Fig. 4f).

### Transcriptomic profile of transition from green leaf to glasshouse bract in noble rhubarb

**Fig. 5:**
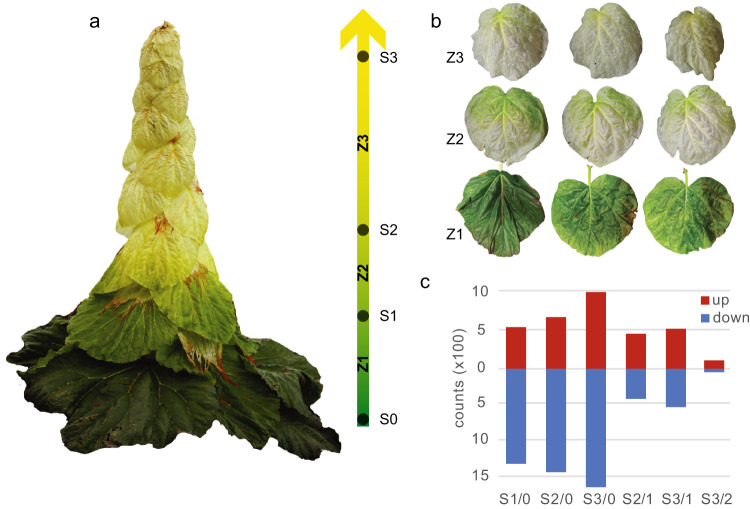
Morphological transition. **a** The leaves were classified into three zones based on their morphology. **b** The phenotype of representative leaves in each zone. **c** Significantly differentially expressed genes (*P* < 0.05, |log_2_(fold-change)| > 1) from different comparisons between leaf types.

A main goal of this research is to decipher the genetic basis of the glasshouse morphology. We focus on the differentiation of the glasshouse bracts from normal leaves using a comparative transcriptomic approach. There are developmental intermediates between green leaves and yellowish bracts (Fig. 5a, b), and the glasshouse-like phenotype starts from where there are flowers and is gradually enhanced upward. We defined the transition from green leaves to glasshouse bracts into three zones: zone 1) from the base of the plant to the base of the raceme, where inflorescences start to develop (Fig. 5b, top); zone 2: from the base of the raceme to where the glasshouse bracts develop; leaves in this zone are green at the base and yellow at the apex (Fig. 5b, middle); and zone 3: the upper section of the plant, where bracts are uniformly yellow (Fig. 5b, bottom). Based on our definition of the transition, we generated transcriptomes from all transition points (S0,1,2,3). The overall expression profiles of the transitions were distinct between each pair, with S2 being close to S3 (Supplementary Fig. 23). Using the basal green leaves (S0) as a comparison, 1849, 2102, and 2635 significantly differentially expressed genes (DEGs, *P* < 0.05, |log_2_(fold change)| > 1) at the transition point S1, S2 and S3 were identified, among which 559, 685, and 1045 were upregulated and 1290, 1417, and 1590 were downregulated (Fig. 5c).

**Fig. 6:**
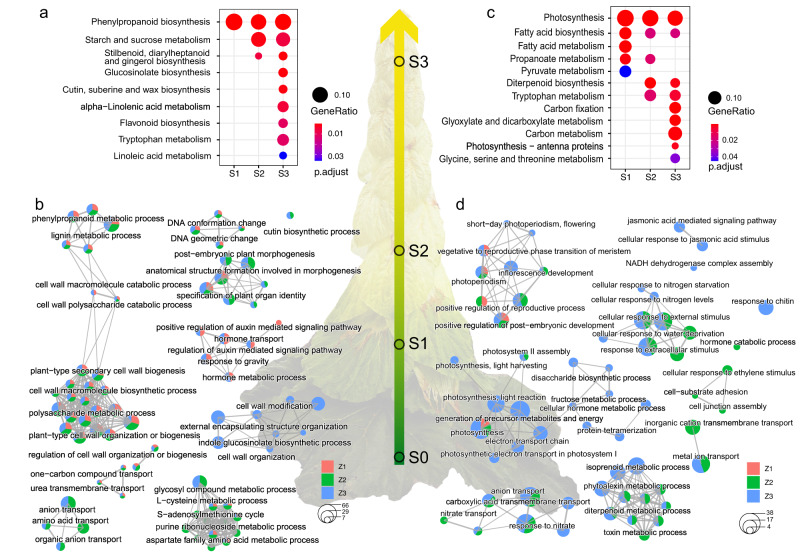
Genetic modules underlying the morphological transition of glasshouse leaves. **a** Enriched KEGG pathways for up-regulated DEGs at three transition points. **b** Network modules of enriched GO terms for up-regulated DEGs. GO terms are linked by similarity matrices calculated using the Jaccard correlation coefficient (JC), and each module is labeled with representative terms selected using REViGO (--Medium, --Arabidopsis, --SimRel) (http://revigo.irb.hr/ accessed at 14-12-2021). **c** Enriched KEGG pathways for down-regulated DEGs. **d** Network modules of enriched GO terms for down-regulated DEGs. The modules are identified in same way as described in **b**.

To identify the genetic modules that are associated with the morphological transition from green leaves to glasshouse bracts, we performed GO/KEGG enrichment analysis for the DEGs identified at transition zones. We first explored the up-regulated DEGs (Fig. 6) and identified nine significantly enriched KEGG pathways (Fig. 6a). Phenylpropanoid biosynthesis was shared by all three zones, and starch and sucrose metabolism and stilbenoid biosynthesis were shared by S2 and S3. The other pathways largely related to specialized metabolites, such as glucosinolate, cutin and flavonoids, were unique in S3 (Fig. 6a).

To further examine which biological processes these DEGs were involved in, we performed GO enrichment analysis. In total, 163 significantly overrepresented GO terms (adjusted *p* ≤ 0.05) were identified in glasshouse bracts as a whole (Supplementary Data 5), and 41 of them were shared by all zones. The enriched GO terms were further grouped into nine network modules (node ≥ 3) (Fig. 6b) by similarity matrices calculated using the Jaccard correlation coefficient^43^. The most notable module was cell wall biogenesis, which included 25 GO terms such as phenylpropanoid metabolic process (GO:0009698), cell wall macromolecule biosynthetic process (GO:0044038), and cell wall biogenesis (GO:0042546). Secondary cell wall biogenesis is the core component of this module as lignin biosynthesis (GO:0009809), xylan biosynthesis (GO:0045492) and glucuronoxylan biosynthesis (GO:0010417) provide polymers for secondary cell wall deposition. This module was shared by all glasshouse zones, representing a common feature of glasshouse bracts as a whole. Nonetheless, as expected from the view of morphological transition, several modules were specific to S2 and/or S3, such as the metabolism of aspartate family amino acids, and amino acids transport (Fig. 6b).

Following the same procedure, we then examined the down-regulated DEGs (Fig. 6c, d). Overall, 12 KEGG pathways were enriched in glasshouses bracts, and two of them (photosynthesis and fatty acid biosynthesis) were shared by all zones (Fig. 6c). In contrast to the up-regulated DEGs which were mainly enriched in specialized metabolism pathways, the down-regulated DEGs were mainly enriched in primary metabolism pathways including fatty acids, amino acids and propanoate metabolism, to name a few (Fig. 6c). The suppression of these pathways in glasshouse bracts, together with S3-specific pathways, such as carbon fixation/metabolism and glyoxylate metabolism, were likely to be a consequence of down-regulation of photosynthesis. Notably, diterpenoid biosynthesis, a specialized metabolism pathway, was significantly enriched among down regulated genes in S2 and S3 (Fig. 6c). GO enrichment analysis identified 60 GO terms for the down-regulated DEGs (Supplementary Data 5), and these GO terms were further grouped into six network modules (node ≥ 3) (Fig. 6d). Four of them were unique to S2 and/or S3, and no module as a whole was shared by all zones (Fig. 6d).

## Discussion

Genomic modification associated with adaptation to high elevations has been well documented in animals^15^ but has been less explored in plants. The rhubarb genus has experienced a rapid evolutionary radiation in Himalayan alpine regions over the past 10 Mya^30,31^, with noble rhubarb inhabiting up to 5000 m in elevation. The rapid radiation of *Rheum* may have been accompanied by hybridization and gene flow between species, resulting in reticulated evolutionary relationships as captured in our phylogenomic analysis. Our comparative genomic analysis provided several insights into this alpine adaptation. Firstly, genes involved in DNA repair, such as homologous recombination and mismatch repair, significantly expanded. As noble rhubarb grows at high elevations, intense UV radiation is a major challenge. UV-B irradiation can cause direct DNA damage^44^ and enhanced DNA repair systems are essential for the survival of noble rhubarb in the alpine environment. For example, homologous recombination is widely used by cells to accurately repair harmful breaks that occur on both strands of DNA^45^. Secondly, genes involved in lipid metabolism and regulation, specifically the unsaturated fatty acids, significantly expanded. As a major component of cell membranes, lipids have a significant role in response to cold stress, both as a mechanical defense through leaf surface protection and plasma membrane remodeling, and as signal transduction molecules^40^. For example, by increasing the level of unsaturated lipids, the plasma membrane can maintain its fluidity and stabilization under low temperature, allowing cells to mechanically adapt to cold^46,47^.

In addition, LTR proliferation has bloated the noble rhubarb genome and promoted expression divergences among duplicated genes, which might contribute to the evolutionary adaptation of noble rhubarb to alpine environments. LTRs are the most important drivers of plant genome evolution^48^ and their proliferation has been demonstrated to be associated with evolutionary innovations in diverse lineages of organisms, including plants^49,50^ and animals^51^. In particular, they can be co-opted to play key organismal functions by providing genes with promoters and enhancers^52,53^, by rewiring regulatory networks^54^, and by assisting the evolution of entirely new genes^48,55^.

Transposable elements have been found to be more active when organisms are under stress, such as in face of changing environments^56^. The uptick of LTR activity in glasshouse plants (1-2 Mya) might be activated by environmental stress caused by Tertiary orogeny, as it coincided with the latest phase of the rapid uplift of the Tibetan Plateau (1.6–3.6 Mya)^57^. Following the initial insertion, the high frequency and efficiency of LTR removal as evidenced by the large numbers of solo LTRs has affected the distribution of LTR. During the removal process, the enrichment of LTR insertions in regulatory regions (<1 kb flanking) and the altered gene expression suggest functional role of LTR insertions and selective retention. These results indicate that the expansions of LTRs in noble rhubarb genome may have rewired the genome-wide gene regulation network. The activity of LTR-retrotransposons fueled by Himalayan geological events may have served as an engine of evolution for plants to adapt to alpine environments.

The specialization of glasshouse bracts to serve as UV filters and warmth-trapping structures may involve complex genetic and physiological alterations. The bracts of *R. nobile* have neither palisade nor spongy parenchyma in their mesophyll but are characterized by highly vacuolated epidermal and hypodermal layers^9^, while that of *R. alexandrae*, another glasshouse plant, have partially differentiated mesophyll cells with intercellular spaces analogous to spongy parenchyma^10^. Both have fewer and malformed chloroplasts compared with normal leaves^9,10^. In general, the anatomical structure of the bracts is similar to that of young leaves at a pre-expansion stage, as undifferentiated mesophyll is a general feature of immature leaves^58^.

Our comparative transcriptomic analyses on the differentiation of glasshouse bracts provided further insights into the development of glasshouse morphology. Firstly, photosynthesis was down regulated in glasshouse bracts indicating the suppression of photosynthesis in glasshouse bracts. This is evident as glasshouse bracts are visibly light yellow, likely due to the absence of the green chlorophyll pigments and fewer and malformed chloroplasts compared to normal leaves^10^. Secondly, secondary cell wall biogenesis appears to be upregulated in glasshouse bracts. Secondary cell walls are typically deposited in specialized cells, such as tracheary elements, fibers and other sclerenchymatous cells, and are in general a small component in leaf tissues^59^. The function of secondary cell walls and cuticle upregulation may relate to the optical property of the tissue. Thirdly, terpenoid metabolism, including diterpenoid metabolic process (GO:0016101) and isoprenoids metabolic process (GO:0006720), was down regulated in glasshouse bracts, probably because of the suppression of photosynthesis.

Finally, the bracts accumulate high levels of flavonoids, which constitute the key feature of its UV filtering function^16^. Our metabolite profiling showed that normal leaves and glasshouse bracts accumulate comparable level of flavonoids in the noble rhubarb, but the latter are differentiated by losing two of the five quercetin-based glycosides and by hyper-accumulating hyperin (quercetin-3-O-galactoside). Flavonoids are well known as photoprotection molecules because of their high efficiency in scavenging UV-induced reactive oxygen species^35^ and have been proposed as one of the key metabolic innovations promoting plant terrestrialization^60^. Flavonoid metabolism is thus an adaptive trait for land plants^61^, and is particularly essential for alpine plants exposed to excess UV radiation. In addition, non-leaf tissues in noble rhubarb contain only traces of flavonoids, suggesting tissue-specific genetic regulation. The presence of glasshouse bracts equipped with UV-absorbing flavonoids and low in chlorophyll that covers the influence thus provides several advantages: 1) they selectively block short-wavelength light to protect reproductive organs from UV damage; 2) they effectively transmit infrared light which has high thermal efficiency, thereby trapping warmth and heating the flowers; and given the benefits mentioned above, 3) they create an optimal thermal microclimate for reproduction, and promote pollination by attracting pollinators, as has been reported in *R. alexandrae*^12^.

Glasshouse morphology is a charismatic plant adaptation in the alpine biome. Although the basic morphological and ecological features of the syndrome have been described^9,10,12,13,30^, to what extent these phenotypes are derived through similar genetic mechanisms are not known. We provided a phylogenetic framework to describe the multiple origins of glasshouse morphology in *Rheum*. The reported unique genomic architecture of *R. nobile* will facilitate further genomic comparisons between glasshouse species and non-glasshouse species. The transcriptomic atlas described here in context of the morphological transition of glasshouse leaves will enable additional comparative analyses to identify the genetic basis of the convergent morphogenesis that has given rise to independent origins of the glasshouse syndrome.

## Methods

### Phylogenetic placement of glasshouse trait

To track the origin and evolution of glasshouse morphology in *Rheum* L., we sampled 17 accessions representing four of the six sections of *Rheum*^7^, together with five species from Polygonaceae as outgroups to build phylogenetic tree. Because of the extensive morphological divergences found in *Rheum*, multiple accessions for the glasshouse plants *R. nobile* and *R. alexandrae* were included in the analysis. Previous phylogenetic inferences^30,62^ with general gene markers, such as ITS and ETS, showed limited power to reconstruct the evolution history of *Rheum*, and we thus employed the phylogenomic approach^63^ to infer the phylogeny of *Rheum*. In total, 22 transcriptomes either from public repositories or generated in this study (Supplementary Data 1) were used in the phylogenomic analysis. Fresh plant tissues were collected in the field, immediately frozen in liquid nitrogen, and were stored in −80 °C until RNA extraction. The voucher specimen was deposited in the Herbarium of Kunming Institute of Botany (KUN). Total RNA was isolated from fresh tissues using the PureLink Plant RNA reagent (Life Technologies) and further purified using TRIzol reagent (Invitrogen). Quality and quantity were examined using a Bioanalyzer 2100 (Agilent Technolo-gies, CA). cDNA libraries with insert sizes 300 bp were constructed using the TruSeq Kit (Il-lumina) and then sequenced as 2 × 150 bp reads on the Illumina HiSeq 1500 platform (Illumina Inc., CA, USA) at BGI (Shenzhen, China). The information of raw reads acquired for each sample is in Supplementary Data 1. Multiple accessions for the glasshouse plant species *R. nobile* and *R. alexandrae* were included. In addition, *Fagopyrum tataricum* which is in the same family as *Rheum* was used as outgroup.

RNA-seq reads, either generated in this study or obtained from public repository (Supplementary Data 1), were first subjected to quality control using FastQC v0.11.8 (https://www.bioinformatics.babraham.ac.uk/projects/fastqc/) and trimming using Trimmomatic v0.39 (HEADCROP:10 LEADING:3 TRAILING:3 SLIDINGWINDOW:4:15 MINLEN:36)^64^. The clean reads were assembled de novo using Trinity v2.3.2^65^ with default settings. CDS sequences were identified and translated using TransDecoder v5.5.0^66^ with homology based ORF (open reading frame) retention criteria (--retain_pfam_hits, -retain_blastp_hits). The homology information was obtained via BLASTp against the plant protein database alluniRefprexp070416^67^ and pfam searches with Pfam 33.1^68^. All translated amino acid sequences were then reduced using CD-HIT-V4.6.1 (-c 0.99 -n 5 -M 60000)^69^. These assembled transcriptomes, together with the CDS sequences from the annotated genome of *F. tataricum*^70^ were used for phylogenomic reconstruction.

Orthology clustering was performed using Orthograph v0.7^71^ with the high-quality orthologous gene clusters of noncore Caryophyllales^72^ as reference. Clusters with at least 70% species occupancy were further used for gene tree inference. Each cluster was aligned using MAFFT v7.407^73^ with default settings, and the alignment was trimmed with Trimal v1.2 (-gt 0.5 -st 0.1)^74^. Phylogenetic trees were estimated using FastTree v2.1.7^75^. Long branches [absolute length (LABS) > 2 or relative length (LREL) > 10)] that were likely introduced by transcriptome assembly artifacts and/or distantly related homologs^63^ were removed. The cleaned clusters were realigned and trimmed following the same procedure. Alignments with species occupancy > 80% and alignment length > 300 were used for further analysis, resulting in 2554 clean orthologous groups of which 187 groups contain only single-copy gene. Phylogenies were reinferred using RAxML-NG v0.7.0b (--model GTR + G --tree_pars 10)^76^. The whole process was pipelined using python scripts adapted from^63^. In addition, we tested the robustness of the phylogeny using different subsets of genes. First, subset of single copy orthologs (one-to-one orthologs) from the 2554 orthologous groups were chosen, which resulted in 187 genes; second, further filtering by average bootstrap values for each gene tree (bs > 90) which resulted in 132 genes; third, based on the filtered gene set, both concatenated- and coalescent-based approach were used to infer the final species tree. Species tree was then constructed using ASTRAL-Pro v5.7.7^77^ based on the single-copy 132 gene trees. Uncertainty for the species tree was estimated using local posterior probability (localPP)^78^. Densitree of *Rheum* was generated from 187 single-copy genes.

To infer the species divergence time, we used the Bayesian clock dating^79^. The topology of the ASTRAL tree and the concatenation of the 132 single-copy gene sequences were used. Two fossils were employed as minimum-age calibrations, including *Polygonocarpum johnsonii* (>66 mya) which calibrates the crown node of Polygonaceae^80,81^ and *Aldrovanda intermediata* (>41.2 mya) which calibrates the crown node of *Aldrovanda* + *Dionaea*^80,82^. The calculations were performed using MCMCTree module (*clock* = 2 *sampfreq* = 1000 *nsample* = 50000000) as implemented in PAML v4.9e^83^.

### Ecological experiments and spectroscopic characterization

To determine the effect of the bracts on the light spectrum reaching the reproductive organs, we measured the reflectance and transmittance spectrum of bracts and leaves using a spectrophotometer. We followed previous studies^84,85^ to set up the equipments (Supplementary Fig. 2). Both transmittance and reflectance spectra were measured using an IdeaOptics PG2000-Pro spectrophotometer (200–1100 nm; IdeaOptics, Shanghai, China) equipped with DH-2000 deuterium-halogen lamp (IdeaOptics). This light source integrates the properties of deuterium lamps and halogen lamps and emits stable UV light (180–400 nm) and visible light (400–750 nm), which coveres the effective light radiation reaching the plants in the wild. The lamps were warmed up for 30 min to obtain stable light emission before measuring. The reflectance spectrum was calibrated with respect to a white Lambertian reflectance standard (STD-WS, IdeaOptics).

Three mature plants with fully developed bracts were randomly selected for measuring in the field (N29.40°-E94.90°, Alt. 4200 m). Bracts from the upper, middle and lower part of each plant were measured separately (Supplementary Figs. 3, 4). For transmittance measurements, the sample was illuminated from outside the sphere directly at an area with 1 mm diameter using optical fiber, and the scattered light was uniformed and detected as described above. For reflectance measurements, the light from the light source was coupled into a 600 μm core-size optical fiber, collimated and illuminated the sample at an 8° angle from within the IS30 integrated sphere (IdeaOptics). The scattered light from the sample was then uniformly diffused within the integrated sphere and collected in another 600 μm core-size optical fiber coupled to the spectrophotometer in 90° angle (Supplementary Fig. 2, inset). To account for the different patterns of light transmittance observed in bracts and leaves, we performed statistical tests. The transmission rate at six wavelengths (250, 350, 450, 550, 650, 750 nm) which cover the UV-visible range were compared using *T* test with three biological replicates.

The ambient temperature and temperature inside the glasshouse-morphology (ca. 100 cm above the ground) were synchronously recorded using a two-channel thermocouple data logger (HOBO-U23-003, Onset, USA) equipped with two alloy needle-type sensor probes (Onset, USA). Temperatures were recorded every 2 minutes in a 48-hour interval at the mid-August in east Himalayas (N29.40°-E94.90°, Alt. 4200).

### Flavonoids extraction and quantification

To profile the flavonoid content in *R. noble*, the flavonoids from different organs (bracts, leaves, stems, fruits, roots and seeds) were extracted using Methanol-Formic Acid solution (75%, 0.1%, both v/v). For each tissue type, three biological replicates, each represented by three technical replicates from the same individual, were used in the measurements. Full steps for the sample preparation are available in protocols.io (https://protocols.io/view/characterization-of-flavonoids-bkekktcw). Apigenin, Quercetin (Aladdin, China) and Isovitexin (Macklin, China) were used as reference standards and were dissolved in 75% MeOH with 0.1% formic acid. The crude extracts were filtered by 0.22 μm PVDF membrane and 10 μL was sampled and separated on an Athena C18 HPLC column (120 Å, 4.6 ×150 mm, 3μm; Anpel Technologies, Shanghai, China) connected to the Access Max HPLC system (Thermo Fisher Scientific). Mobile phases of 0.5% (v/v) formic acid in deionized water (solvent A) and 0.5% formic acid in acetonitrile (solvent B) were used at a flow rate of 600 μL/min. The gradient program started at 10% B which was increased linearly to 20% in 6 min and to 30% in 20 min; then eluent B was held constant for three minutes at 90% and linearly decreased to 10% in two minutes and was held constant for one minute. Finally, the column was equilibrated for 10 minutes at the initial solvent composition.

Mass spectrometry was performed using a 6546 Q-TOF LC-MS-MS system from Agilent Technologies (Santa Clara, CA, USA) coupled with an electrospray ionization (ESI) interface. The parameters were optimized as follows: ESI voltage −4,000 V, nebulizer gas 60, auxiliary gas 50, curtain gas 35, turbo gas temperature 500 °C, declustering potential −60 V, and focusing potential −350 V. The samples were analyzed with an information-dependent acquisition (IDA) method, which can automatically select candidate ions for the MS-MS analysis. The TOF mass range was set from m/z 50 to 800, and the mass range for product ion scan was m/z 50–800. The collision energy was set to 10 eV to observe the pseudo-molecular [M-H]^-^ ion and the losses of substituent groups, and 20–40 eV to obtain information about the basic skeletons. The mass analyzer was calibrated using Taurocholic acid (2 ng/μL) by direct injection at a flow rate of 5 μL/min. Under the negative ESI mode, molecules with phenol hydroxyl could produce strong and stable [M-H]^-^, which could be helpful for identification^86^. The ESI-MS^n^ spectrum for each isolated compound (Supplementary Figs. 5–10) was searched against the NIST/EPA/NIH mass spectral library as supplemented in Xcalibur v4.3 (Thermo Scientific, MA, USA), and was also compared with that of reference compounds generated under the same LC-MS conditions. The most abundant flavonoid molecule (peak 3, Supplementary Fig. 5) produced a deprotonated ion at *m*/*z* 477 in the ESI-MS^1^ spectrum (Supplementary Fig. 6). In the ESI-MS^2^ spectrum, two high intensity fragments at *m*/*z* 301 [M-H-162]^–^ and 300 [M-H-162] ^–•^ were observed. Ions at *m*/*z* 301 suggested the loss of a hexose unit. Ions at *m*/*z* 271, 255, 179, 151 and 121, obtained with CE = 40 eV, were the characteristic ion products of quercetin. The higher intensity of [M-H-162] ^–•^ than that of [M-H-162] ^–^ led to the identification of a glycosylation site at 3-OH. On the basis of the mass spectra and co-chromatography with an authentic standard, this compound was tentatively identified as quercetin-3-O-galactoside (Supplementary Fig. 6). Similarly, the other compounds were identified as quercetin 3-O-rutinoside (peak 1), quercetin 3-O-glucoside (peak 2), quercetin 3-O-arabinopyranoside (peak 4,) and quercetin 3-O-[6´´- (3-hydroxy-3-methylglutaroyl)-glucoside] (peak 5) (Supplementary Figs. 7–10). The main components from different tissues were quantitatively characterized using the internal standard method in HPLC with apigenin and quercetin as standards. Three biological replicates for each tissue type were used in the quantitative analysis.

### Plant materials and genome sequencing

Plant tissues (leaves, bracts, fruits, stems, roots) of *R. nobile* were collected from a single individual in the Huluhai, Hengdun Mountains (N28.53°-E99.95°; Alt. 4500 m; Yunnan Province, China). Tissues were immediately frozen in liquid nitrogen. The voucher specimen was deposited in the Herbarium of Kunming Institute of Botany (KUN, Deng201901, ZHJ8-1). Total genomic DNA was extracted from young leaves using the Plant DNAzol reagent (Life Technologies, CA, USA) following the manufacturer’s protocols. Quality and quantity were determined by gel electrophoresis and NanoDrop D2000 (Thermo Scientific, Waltham, USA). Paired-end libraries with insert sizes of 270 bp and 500 bp were constructed and sequenced as 2×150 bp reads on the Illumina X Ten system at BGI (Shenzhen, China). Single Molecule, Real-Time (SMRT) sequencing techniques were used to generate long reads. DNA libraries with 20 kb inserts were constructed and sequenced on PacBio Sequel system at BGI (Shenzhen, China) according to the manufacturer’s protocols. To facilitate genome annotation, we performed transcriptome sequencing using different tissues (bract, leaf, stem, fruit, root). Total RNA was isolated from fresh tissues using the PureLink Plant RNA reagent (Life Technologies) and further purified using TRIzol reagent (Invitrogen). Quality and quantity were examined using a Bioanalyzer 2100 (Agilent Technologies, CA). cDNA libraries with insert sizes 300 bp were constructed using the TruSeq Kit (Illumina) and then sequenced as 2 × 150 bp reads on the Illumina HiSeq 1500 platform (Illumina Inc., CA, USA) at BGI (Shenzhen, China).

Illumina raw reads were assessed by FastQC v0.11.8 (https://www.bioinformatics.babra-ham.ac.uk/projects/fastqc/) and trimmed using Trimmomatic v0.39 (HEADCROP:10 LEAD-ING:3 TRAILING:3 SLIDINGWINDOW:4:15 MINLEN:36) to generate the final clean reads (Supplementary Data 2).

PacBio subreads were obtained using the SMRT Analysis RS.Subreads.1 pipeline (minimum polymerase read quality = 0.80; minimum polymerase read length and minimum subread length = 50). In total 12.9 million subreads (116.35 Gb) were generated with an N50 of 13.3 kbp, which result in 83× coverage based on the genome size estimated via k-mer distribution using Illumina reads.

### Genome assembly, gene prediction and functional annotation

Genome features such as genome size, repeat content and heterozygosity were characterized with 49 Gb of high-quality Illumina paired-end reads using K-mer distribution. 86 Gb high-quality Illumina paired-end reads were extracted from the 147.6 Gbp clean reads using Trimmomatic v0.39 (HEADCROP:20 CROP:100 LEADING:3 TRAILING:3 SLIDINGWINDOW:4:15 MINLEN:100), and then were used to generate the frequency distribution of k-mer (k = 19) using Jellyfish v2.3.0 (-C -m 19)^87^. The genome features were estimated by GenomeScope v1.0.0^88^ with high-frequency k-mer (>1k) excluded.

We used PacBio long reads to generate a high continuity assembly which was subsequently polished with Illumina short reads. The overall assembly workflow for noble rhubarb is shown in Supplementary Fig. 12. Because the genome is highly repetitive, we used the PacBio long reads for a de novo genome assembly and used the highly accurate Illumina short reads for a final polishing. PacBio subreads were first corrected by Canu v1.7.1^89^ and then subjected to different long read assemblers (Canu, Falcon, Flye, miniasm). Because of the high proportion of repetitive sequences in the genome, we excluded reads shorter than 10 kb from the assembly processes. The final assembly was generated based on corrected reads longer than 10 kb (5.5 million reads, 69-fold coverage) using Canu v1.7.1 with the following parameters: genomeSize=1300 m, corMhapFilterThreshold=0.0000000002, ovlMerThreshold=500, corMhapOptions = ‘--threshold 0.8 --num-hashes 512 --num-min-matches 3 --ordered-sketch-size 1000 --ordered-kmer-size 14 --min-olap-length 2000 --repeat-idf-scale 50’. This assembly contains 940 contigs with N50 = 5.6 Mbp and is superior to the results of other assembly approaches.

To polish the assembled sequences, we employed an iterative approach to correct errors (Supplementary Fig. 12). The PacBio long reads were mapped to the assembly using pbmm2 v1.3.0 (https://github.com/PacificBiosciences/pbmm2) with default settings. Arrow, from the package GenomicConsensus v2.3.3 (https://github.com/PacificBiosciences/Genomic Consensus/releases/tag/2.3.3), was then applied twice to correct the contigs based on the PacBio reads. The corrected sequences were then polished with Pilon v1.23^90^ using the more accurate Illumina short reads that were mapped to the contig sequences using BWA-MEM v0.7.13^91^ with the -M option to flag spurious alignments. This Pilon correction was repeated three times.

The overall heterozygosity of *R. noble* genome is low (0.0576%) (Supplementary Fig. 13), but regions with high heterozygosity may be assembled as separate allelic contigs rather than single haplotype-fused contig. To account for this artificial regional duplication, we used *purge_haplotigs* pipeline^92^ to sort out the redundant allelic contigs. Using the polished assembly as reference, we first mapped the 86 Gb high-quality Illumina paired-end reads using samtools v1.14^93^ and Minimap2 v2.17^94^. We then used *purge_haplotigs* to 1) obtain the read-depth histogram from the mapped BAM files (*purge_haplotigs readhist alignment.bam*) and estimate the cutoffs for low coverage, low point between the two peaks, and high coverage; 2) identify allelic contigs based on the cutoffs estimated in step 1 (*purge_haplotigs contigcov -i aligned.bam.genecov -l 20 -m 45 -h 120 -o coverage_stats.csv*); 3) reassign the homologous contigs and output a haploid assembly (*purge_haplotigs purge -g genome.fasta -c coverage_stats.csv -b aligned.bam*). After these process, 70.58 Mb sequences were identified as redundant allelic contigs and were removed from the assembly.

We assessed the completeness and contiguity of the final assembly using various approaches (Supplementary Figs. 14, 15). First, we used QUAST v5.0.2^95^ to generate the general assembly statistics (Supplementary Data 2). The final assembly is 1,362,557,596 bp with 245 contigs, making up more than 91.9% of the genome based on the estimated genome size. Second, we mapped 492 million paired-end short reads to the assembly using Bowtie2 v2.2.6^96^. In total, 99.37% of them were mapped and 98.04% were concordantly paired. We also mapped 13.7 million PacBio long reads to the assembled contigs. Overall, 85.9% were mapped with the longest mapped read being 67,473 bp. We also mapped the RNAseq reads generated from different tissues to the assembly using HISAT2 v2.1.0^97^. The overall alignment rate ranges from 94.66% to 96.24%, and 71.08%-86.30% of the reads aligned concordantly exactly one time. Furthermore, we used BUSCO v3.0.2^98^ with the Eudicotyledons_odb10 dataset to assess the integrity of the assembly. Of the 2121 BUSCOs (Benchmarking Universal Single-copy Orthologs) that shared among Eudicotyledons, 96.1% were recovered with 87.3% being single-copy (Supplementary Fig. 14). In addition, we also employed the LAI (LTR Assembly Index)^99^ to assess the continuity of the assembly. To calculate LAI, the LTRs were first identified by LTRharvest (-minlenltr 100 -maxlenltr 7000 -mintsd 4 -maxtsd 6 -motif TGCA -motifmis 1)^100^, ltrFinder (-w 2 -C -D 15000 -d 1000 -L 7000 -l 100 -p 20 -M 0.85)^101^ and ltrDetector (default settings)^102^. Intact LTRs (LTR retrotransposons with perfect micro-structures of terminal motifs and target sites) were obtained using ltrRetriever^103^. 31705 clean LTRs were identified and 10619 of them were intact. All LTR sequences were then annotated by RepeatMasker v4.0.9 using the non-redundant LTR-RT library constructed by LTR_retriever. The LAI was then calculated by the LAI module as implemented in ltrRetriever. Finally, we used Merqury^36^ to estimate consensus quality value (QV) and k-mer completeness of the assembly. First, 71.1Gbp Illumina reads (~48 fold of estimated haploid genome size) were used to generate a 19-mer meryl database. Then the QV and k-mer completeness were inferred by the comparing k-mers of the assembly to that of the unassembled Illumina reads. The calculations were performed using Merquary v1.3. The QV is 46.3 with an error rate being 2.33471e-05 and estimated assembly k-mer completeness is 99.24%.

To annotate the genome assembly, repetitive sequences were first identified and soft-masked. We used an integrated strategy combining homology-based searches and ab initio searches to identify repeat elements in the genome assembly (Supplementary Fig. 16). In short, transposable elements (TE) were predicted by RepeatModeler v2.0.1^104^. TE sequences were searched against the plant protein database RefSeq (ftp://ftp.ncbi.nih.gov/refseq/release/plant/) using ProtExcluder v1.2 (http://weatherby.genetics.utah.edu/MAKER/wiki/index.php/Repeat_Library_Construction-Advanced). Elements with a significant hit to plant genes, along with 50 bp upstream and downstream of the BLAST hit were removed. The identified TE sequences, together with Dfam v3.1^105^ and RepBase v20170127^106^ were then used as repeat libraries for RepeatMasker v4.0.9 (http://www.repeatmasker.org) for homology based repeat identification.

Ab initio prediction was performed following the BRAKER2 pipeline^107^. In brief, soft masked genome sequences and spliced RNA-seq alignment information were used to train GeneMark-ET^108^ and generate the *R. noble* rhubarb specific gene model parameters. These specific parameters, together with the spliced alignment information were subjected to AUGUSTUS v3.2.1^109^ to identify gene models. Homology based predictions were conducted using GeMoMa v1.6.1^110^ with protein sequences of Tartary buckwheat^70^ and sugar beet^38^ as hints. The homology-based gene models were then combined, and low-quality and redundant gene models were removed. To finalize the gene models, all predictions, together with *R. noble* transcripts generated as mentioned above, were integrated using EVidenceModeler v1.1.1^111^. The final prediction was further updated by PASA v2.4.1^112^ with gene structure information and alternatively spliced isoforms incorporated.

The predicted gene models were searched against NCBI RefSeq non-redundant proteins (NR database, ftp://ftp.ncbi.nih.gov/blast/db) using DIAMOND v0.9.28 (--outfmt 5 --max-target-seqs 3 --unal 1 --more-sensitive --comp-based-stats 1)^113^. Motifs and domains were searched against the following database: CDD-3.17^114^, Coils-2.2.1^115^, Gene3D-4.2.0^116^, Hamap-2019_01^117^, MobiDBLite-2.0^118^, PANTHER-14.1^119^, Pfam-32.0^68^, PIRSF-3.02^120^, PRINTS-42.0^121^, ProSitePatterns-2019_01^122^, SFLD-4^123^, SMART-7.1^124^, SUPERFAMILY-1.75^125^, TIGRFAM-15.0^126^, and TMHMM-2.0c^127^ using InterProScan v5.40^128^ with default settings. The BLASTp hits and InterProScan results were then imported into Blast2Go v5.2^129^ for GO mapping and functional assignment.

Non-coding RNAs (ncRNAs) were annotated using various databases and software packages: Transfer RNAs (tRNAs) were predicted using tRNAscan-SE v2.0.7^130^ with default settings. Ribosomal RNAs (rRNAs) were predicted using RNAmmer v1.2 (-S euk -m lsu,ssu,tsu)^131^. MicroRNAs (miRNAs) and small nuclear RNA (snRNA) were identified by searching the Rfam v14.1 database^132^ using BLASTn v2.2.31 (-W 7 -e 1 -v 10000 -b 10000)^133^ and INFERNAL v1.1.3^134^ with default parameters.

### Comparative genomic analysis

*R. noble* and eight other Caryophyllales species with publicly available high-quality genome sequences (Supplementary Data 3) were included in the analysis. For each of the genome assemblies, we first reduced alternatively spliced transcripts to the longest one per gene using in-house scripts. Colinear gene pairs were identified using DupGen_finder (https://github.com/qiao-xin/DupGen_finder) according to the information of gene similarity and synteny. In brief, for each genome, an all-vs-all BLAST search was performed using BLASTp (-evalue 1e-10 -max_target_seqs 5 -outfmt 6)^133^. Collinear blocks were then identified using MCScanX (-s 5 -e 1e-5 -w 5)^135^. Synonymous substitutions per synonymous site (Ks) of colinear gene pairs were inferred using the NG algorithm^136^ as incorporated in KaKs_Calculator v2.0^137^. Ks values exceeding 5 were excluded from further analysis due to likely substitution saturation^138^. The probability density distribution of the Ks value was fitted with Gaussian mixture models (GMMs) with a coefficient of determination (R^2^) > 0.95.

To further examine the WGD events shared by *R. nobile* and *F. tataricum*, we employed the probabilistic approach using MAPS^139^. MAPS reconciles gene phylogenies to given species phylogeny to infer genome polyploidization events shared by descendants. Because MAPS algorithm works best with simple, ladderized species trees^139^, we selected four species (*R. nobile*, *F. tataricum*, *A. vesiculosa* and *B. vulgaris*) to conduct the probabilistic modeling, which can minimize the noise from divergent phylogenetic relationships and simplify the computation as well. We used OrthoFinder v2.3.12 (-I 1.5 -M msa -A mafft -T FastTree)^140^ to sort out the gene trees. We recovered 5233 gene trees with at least one gene copy from each species and at least 100 amino acids in the alignment. MASP identified 59.5% of the subtrees supporting a shared genome duplication shared by *R. nobile* and *F. tataricum*, and this was significantly higher than that from null model which assumes there is no WGD across the phylogeny (Supplementary Fig. 17b). To distinguish whole genome duplication (WGD) from whole genome triplication (WGT) events, we further conducted pairwise genome synteny analyses using the beet genome (*Beta vulgaris*) as reference^38^. Beet is known to have no more genome polyploidization after the core-eudicots shared gamma event^38^. We calculated the syntenic depth of orthologous genic blocks using Mcscan (https://github.com/tanghaibao/jcvi/wiki/MCscan-(Python-version) and the pattern of syntenic depth (number of *R. nobile* and *F. tataricum* gene blocks per beet gene) was summarized (Supplementary Fig. 18). In addition, we built up the overall genome synteny (Supplementary Fig. 19) between *B. vulgaris* and *R. nobile*/*F. tataricum* using GENESPACE v0.9^141^.

To explore gene family evolution in association with the adaptation of *R. nobile* to alpine environments, we analyzed gene family expansion and contraction in the context of the diversification of Caryophyllales. Protein sequences from the comparative genomic dataset that included nine Caryophyllales genome assemblies and a grape genome assembly (Supplementary Data 3) were first cleaned by removing alternative splicing transcripts (the longest one was retained) and then subjected to OrthoFinder v2.3.12 (-I 1.5 -M msa -A mafft -T iqtree) for orthologous group identification. In total, 376,774 genes (88.1% of total) were assigned to 28,668 orthologous groups. Fifty percent of all genes were in orthologous groups with 19 or more genes (G50 = 19) and were contained in the largest 6138 orthologous groups (O50 = 6138). There were 7144 orthologous groups with all species present and 87 of these consisted entirely of single-copy genes.

The 87 single-copy genes were then used to infer the species tree. Peptide sequences of each single-copy gene were aligned using MAFFT v7.407^73^ with default settings, and the alignment was trimmed with Trimal v1.2 (-gt 0.5 -st 0.1)^74^. The cleaned alignments were then concatenated to a super-matrix and the phylogeny was estimated using RAxML-NG v0.7.0b (--model PROTCAT --tree_pars 10 --bs-trees 200)^76^. After confirming that the species tree topology was consistent with that inferred from much denser sampling^63^, the resulting species tree was used for gene family contraction and expansion analysis. An ultra-metric tree was then generated using r8s v1.5^142^ with dating point (107 Mya) at the split between core and noncore Caryophyllales^143^. The gene family sizes at each internal node were inferred using CAFE v4.2.1^144^ with a maximum-likelihood model. Gene families with ≥ 200 gene copies in one or more species were excluded as large gene copy number variation can cause parameter estimates to be non-informative^144^.

The biological function of these expanded/contracted gene families were explored by GO and KEGG enrichment analyses. The significantly overrepresented GO terms were clustered to reduce redundancy using REViGO (http://revigo.irb.hr/, accessed at 26-11-2021) which summarizes the GO terms based on semantic similarity algorithms^145^.

### Evolutionary dynamics of LTR

Long terminal repeat (LTR) retrotransposons were identified by searching the genome sequences of *R. nobile* using LTRharvest (-minlenltr 100 -maxlenltr 7000 -mintsd 4 -maxtsd 6 -motif TGCA -motifmis 1)^100^, ltrFinder (-w 2 -C -D 15000 -d 1000 -L 7000 -l 100 -p 20 -M 0.85)^101^ and ltrDetector (default settings)^102^. The resulted LTRs were filtered and combined by ltrRetriever^103^ with default settings. 10619 and 17451 intact LTRs were identified from *R. nobile* and *R. hastalutus* genome assembly, respectively (Supplementary Fig. 20). Solo LTRs that are derived from interelement recombination were also identified by ltrRetriever^103^.

To estimate the insertion date of the LTR retrotransposons, we followed the approach described in SanMiguel et al. (1998)^146^. Since the 5’ and 3’ repeat pair of a complete retrotransposon was identical upon insertion, the insertion time can be estimated based on the divergence between the LTR repeat pairs^147,148^. The divergence time (T) between the LTR repeat pairs can be estimated by *K*/2*μ *where *K* is the genetic distance and *μ* is the neutral mutation rate^149^. *K* is estimated by the Jukes-Cantor model for non-coding sequences as

1 $$-\frac{3}{4}{{{{\mathrm{ln}}}}}\, \left(1-\frac{4d}{3}\right)$$

where *d* is the proportion of sequence difference^150^. The speciation time between noble rhubarb and Tartary buckwheat was about 62 Mya^80^. The mean Ks values between noble rhubarb and Tartary buckwheat orthologs was inferred as 0.75 by applying the mixture model of Gaussian distributions to the raw Ks distributions. The number of substitutions per synonymous site per year was thus estimated as *μ* = Ks/2 T = 6.0 × 10^−9^. Using this rate of neutral nucleotide substitutions *μ*, we estimated the insertion time of LTRs in *R. nobile* genome:

2 $${{{{{\rm{T}}}}}}=K/2\mu =-\frac{3}{4}{{{{\mathrm{ln}}}}}\, \left(1-\frac{4d}{3}\right)/2\mu$$

To explore the association of LTR insertions and genes in terms of their physical distance in chromosome, we mapped the LTR insertions onto the genome assembly with the 58,950 annotated gene models as anchors using the *bedmap* tool (--range 5000) as implemented in BEDOPS v2.4.40^151^. An LTR was defined to be associated with gene if it inserted within 5-kb upstream or downstream of coding sequence, or inside protein coding region. If an LTR is shared by more than one gene, only one type is counted with the order: geneic, 5’-upstream and 3’-downstream.

To explore the consequences of LTR expansion, we used the gene pairs that were derived from recent gene duplication events (Ks <0.5) as probe to explore how the expression of genes was affected by the LTR insertions. Comparisons were made between gene pairs that have at least one LTR insertion within 1-kb upstream and no LTR insertion within 1-kb upstream. The divergences in gene expression were determined using Euclidian distance of the gene expression profiles of gene pairs in five organs (leaf, bract, stem, fruit and root). Tissue specificity divergence was calculated based on changes of the *tau*-index^152^ between gene pairs. The calculations were based on log_2_ transformed RPKM values.

### Transcriptomic profiling for the morphological transition in glasshouse morphology

To explore the transcriptomic modification along with the development of glasshouse morphology, we focused on the morphological transition from green leaves to yellowish bracts. We sampled the leaves at four spatial sites representing morphological/physiological transition from normal green leaves to specialized yellowish bracts (Fig. 5a, b). For each sample site, three biological replicates (individual plants) were used and 8-10 discs per leaf were sampled to form a replicate. In total, 12 leaf samples were collected in the wild and frozen in liquid nitrogen immediately.

Gene expression profiles in different samples were generated following the HISAT-StringTie pipeline^153^. Specifically, the cleaned reads were mapped to the genome assembly using HISAT2 v2.1.0^97^ with default settings. The transcripts for each sample were generated using StringTie v2.0.5^153^ with default settings and then merged (StringTie --merge). The final transcript counts for each sample were generated with StringTie (-e -B), and then were analyzed using DESeq2 v1.30^154^. To account for the sequencing depth and RNA composition, the read counts were normalized based on sample-specific size factors determined by median ratio of gene counts relative to geometric mean per gene, allowing for the comparisons between samples. A multidimensional scaling (MDS) plot was generated to show the effect of the batch adjustment across the samples at different time points. Differentially expressed genes (DEGs) between the samples were determined by false discovery rate (FDR) cut-off (*p* < 0.05) and a log_2_ fold-change cut-off (log_2_FC > 1).

Overrepresented GO (Gene Ontology) and KEGG (Kyoto Encyclopedia of Genes and Genomes) terms in target gene sets were identified using Fisher’s exact test^155^, and the significance of the test (adjusted *p* value) were estimated using Benjamini-Hochberg method^156^ by accounting for multiple tests. To perform the enrichment analyses, the gene models were first fully annotated using eggNOG-Mapper (http://eggnog-mapper.embl.de/), and then a custom species-specific database that links gene ID with GO and KEGG terms was built. The overrepresented GO and KEGG terms in gene sets of particular interests, namely the differentially expressed genes, were explored using clusterProfiler v4.0^43^. GO and KEGG enrichment analysis were performed using *compareCluster* (*p*valueCutoff = 0.05, *q*valueCutoff = 0.05) in the R package clusterProfiler.

### Statistics and reproducibility

For spectroscopic measurement and RNAseq, three biological repeats were used. For phytochemical analysis, three biological replicates, each represented by three technical replicates from the same individual were used. Fisher’s exact test and Wilcoxon rank sum test were performed using R v.4.0.2 in RSTUDIO v.1.1.456.

### Reporting summary

Further information on research design is available in the Nature Portfolio Reporting Summary linked to this article.

## Supplementary information

Supplementary Information

Description of Additional Supplementary Files

Supplementary Data 1-5

Supplementary Data 6

Reporting Summary

## Data Availability

Sequencing reads are available under the project CNP0001526 in China National GeneBank DataBase (CNGBdb) with accession number: CNR0350651-CNR0350660 (PacBio reads); CNR0350673-CNR0350676 (Illumina reads); CNR0350677-CNR0350686 (RNA-seq reads). The source data behind the graphs in Figs. 2–6 are available in Supplementary Data 6.
