# Supplementary material for: The genome of the glasshouse plant noble rhubarb (Rheum nobile) provides a window into alpine adaptation
Source: Commun Biol. 2023 Jul 10;6:706. doi: 10.1038/s42003-023-05044-1 (PMC10333194; doi:10.1038/s42003-023-05044-1)
Supplement: Supplementary file 1 — Supplementary Information [file 42003_2023_5044_MOESM1_ESM.pdf]

Supplementary Figures

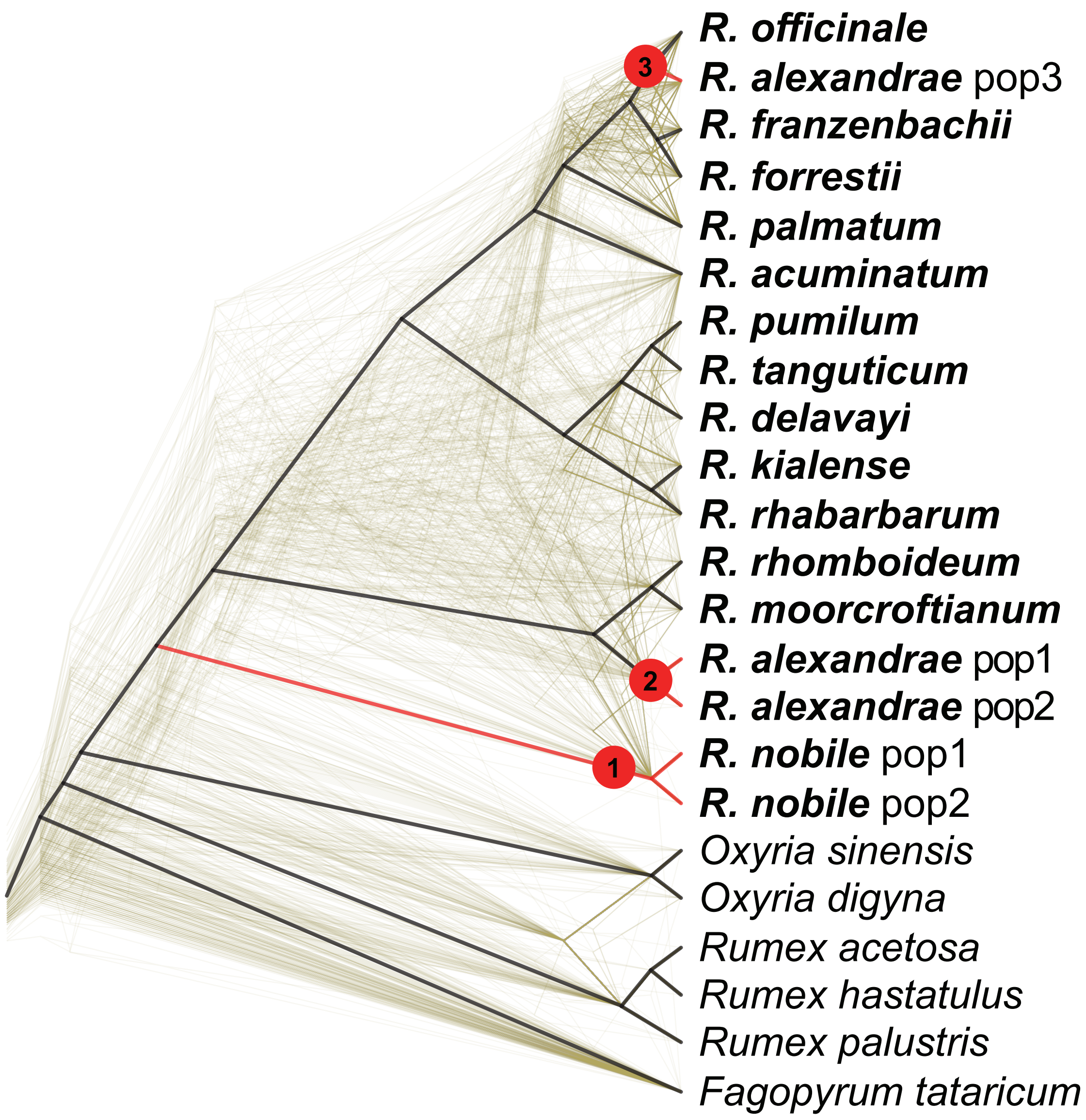

Supplementary Figure 1. Astral coalescent tree of Rheum estimated from 2554 orthologs, with densitree generated from maximum likelihood phylogeny of 187 single copy genes. The glasshouse lineages are labeled in red, and the independent origins of glasshouse syndrome were represented by red dots.

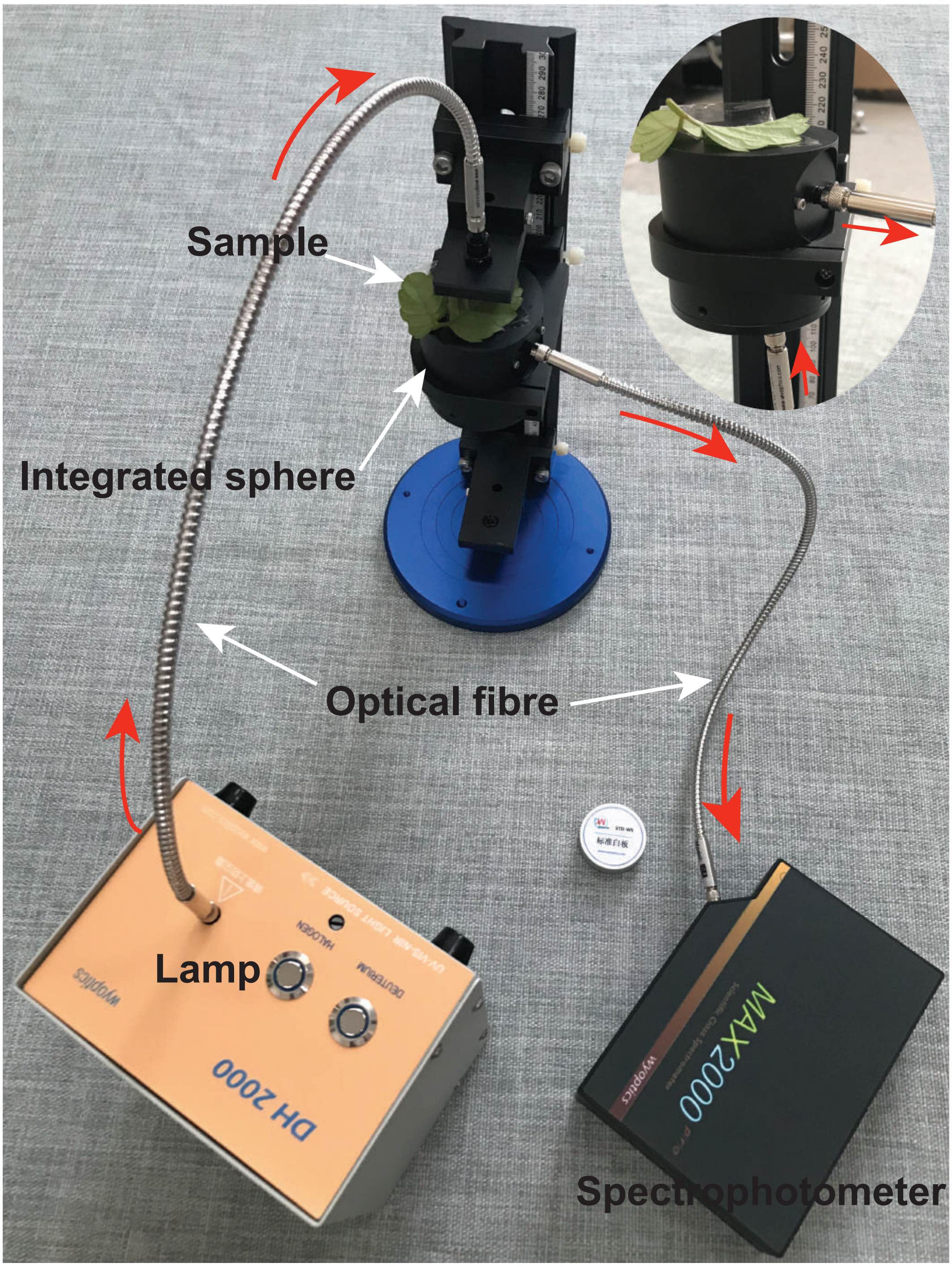

Supplementary Figure 2. The spectroscopic system used to measure the transmittance spectrum and reflectance spectrum (inset) of noble rhubarb bracts and leaves. The direction of the light influx is labeled in red arrows. The measurements were performed in dark environment to eliminate the effects of ambient light.

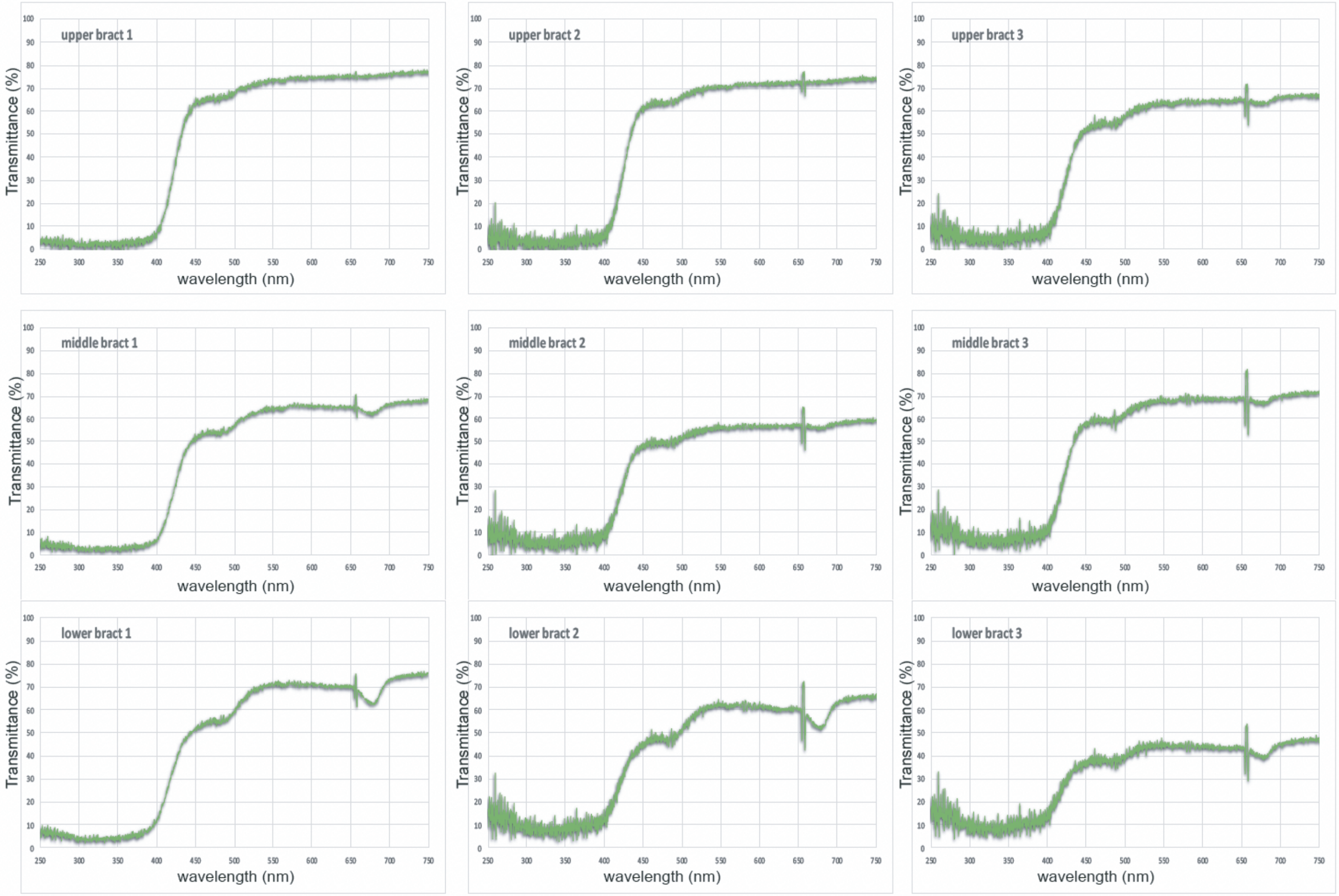

Supplementary Figure 3. Transmittance spectra of ultraviolet and visible light by upper, middle and lower bracts of noble rhubarb.

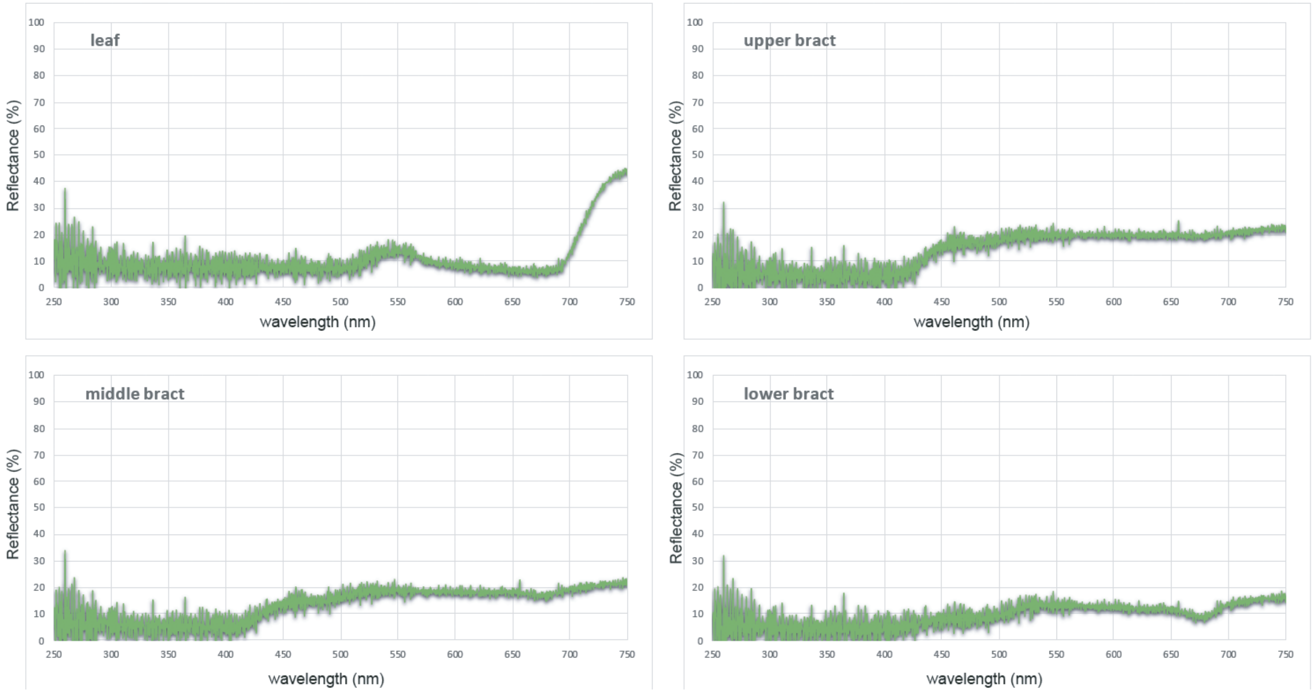

Supplementary Figure 4. Reflectance spectrum of ultraviolet and visible light by leaf and bract (upper, middle and lower) of noble rhubarb.

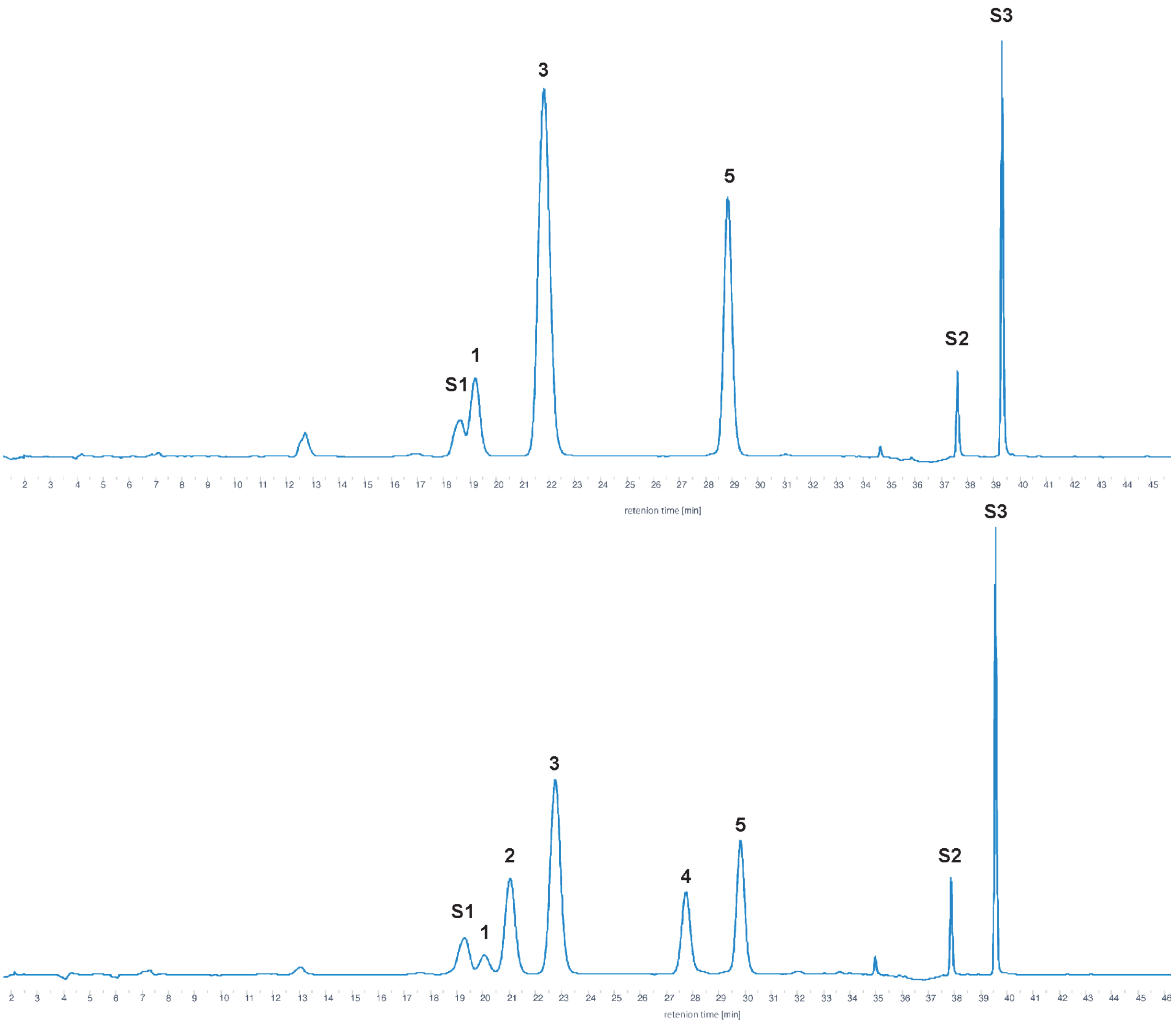

Supplementary Figure 5. The UV trace (350nm) for the LC-MS spectrum of the bract (upper) and leaf (lower) methanol extracts. Flavonoid standards are labeled with S1-S3, and the flavonoid compounds isolated from noble rhubarb samples are labeled with number 1-5.

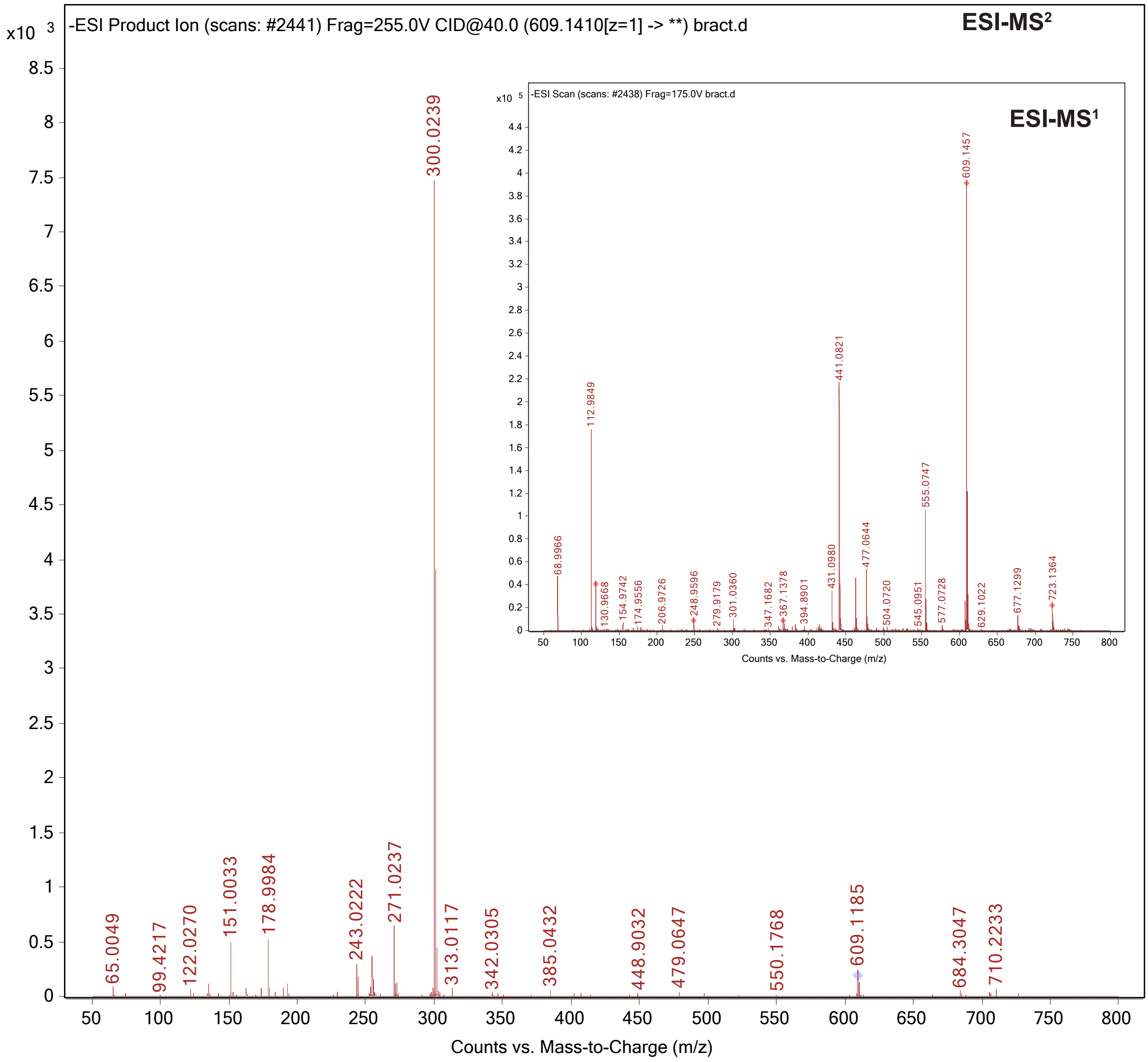

Supplementary Figure 6. The mass race (ES-) of LC-MS spectrum of compound 1 (Supplementary Figure 5) at 40 and 10 eV.

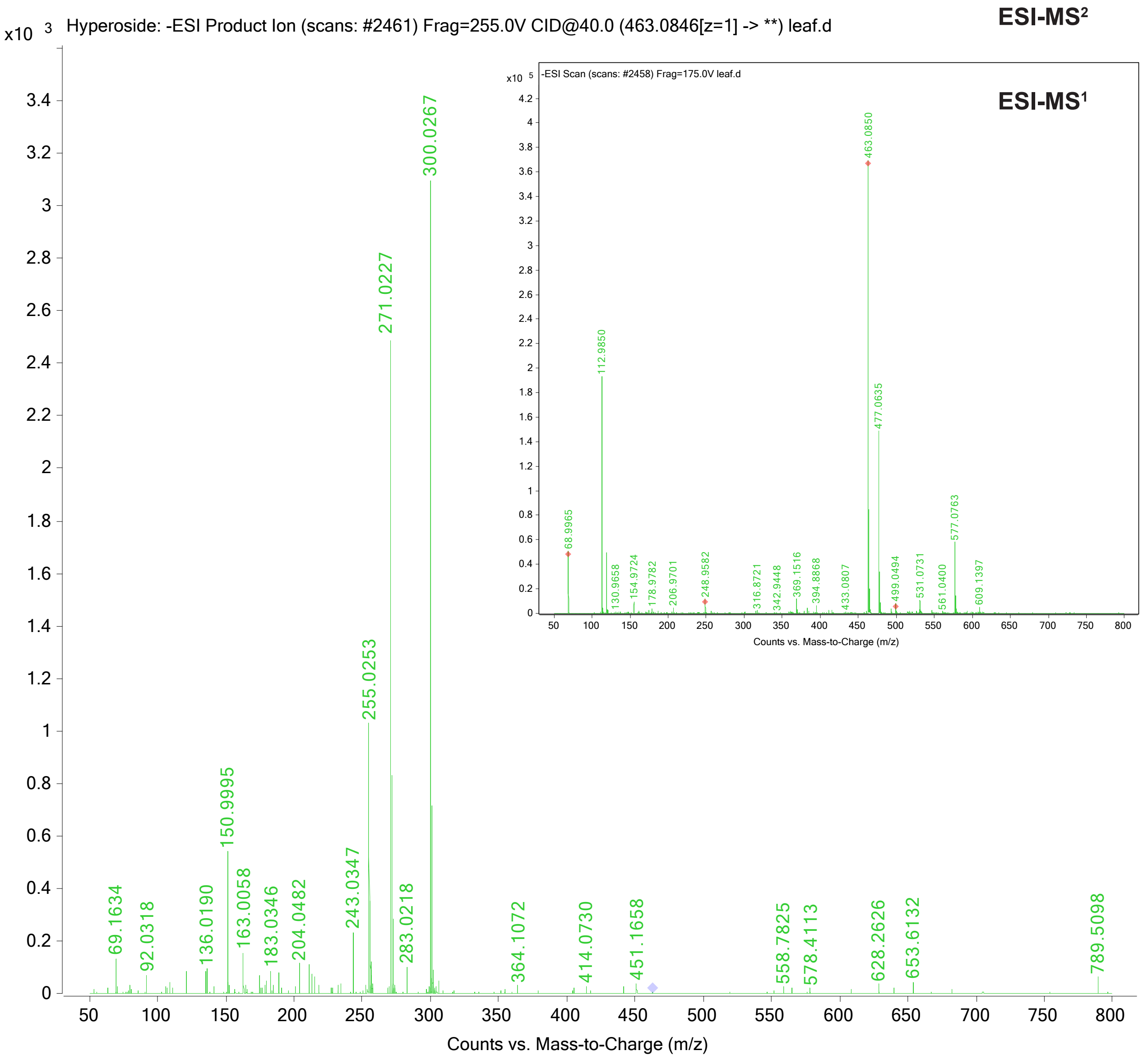

Supplementary Figure 7. The mass race (ES-) of LC-MS spectrum of compound 2 (Supplementary Figure 5) at 40 and 10 eV.

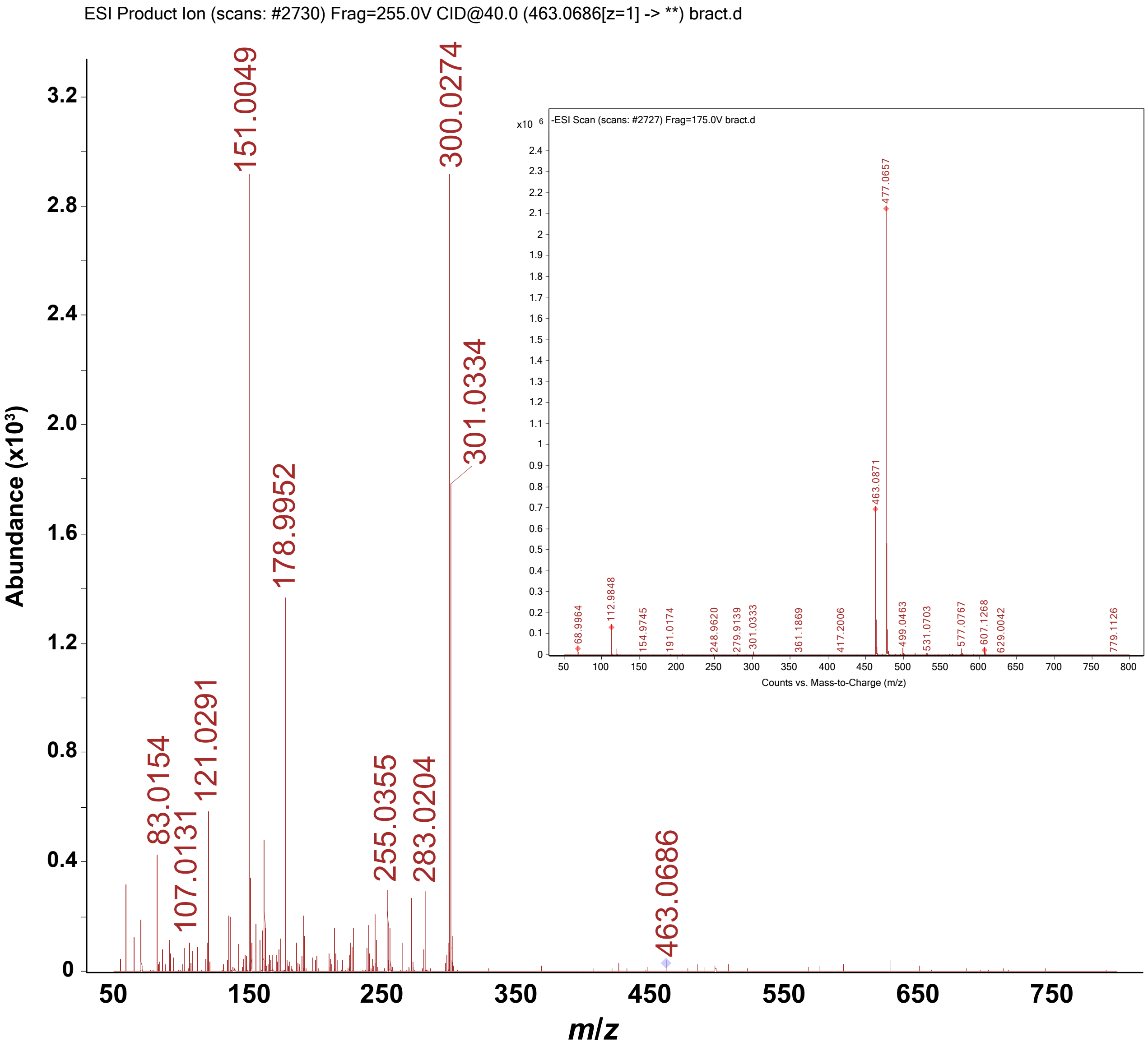

Supplementary Figure 8. The mass race (ES-) of LC-MS spectrum of compound 3 (Supplementary Figure 5) at 40 and 10 eV.

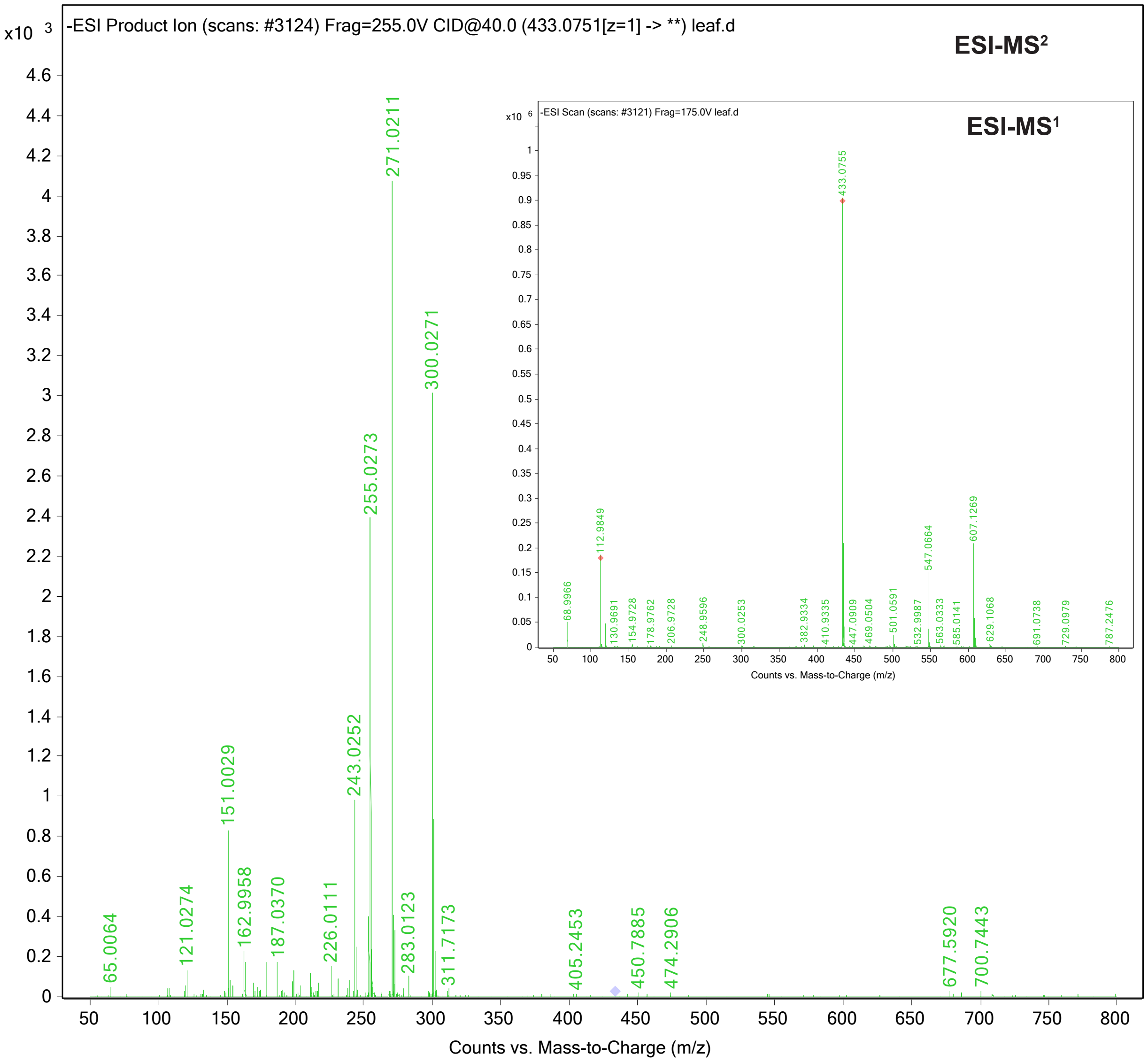

Supplementary Figure 9. The mass race (ES-) of LC-MS spectrum of compound 4 (Supplementary Figure 5) at 40 and 10 eV.

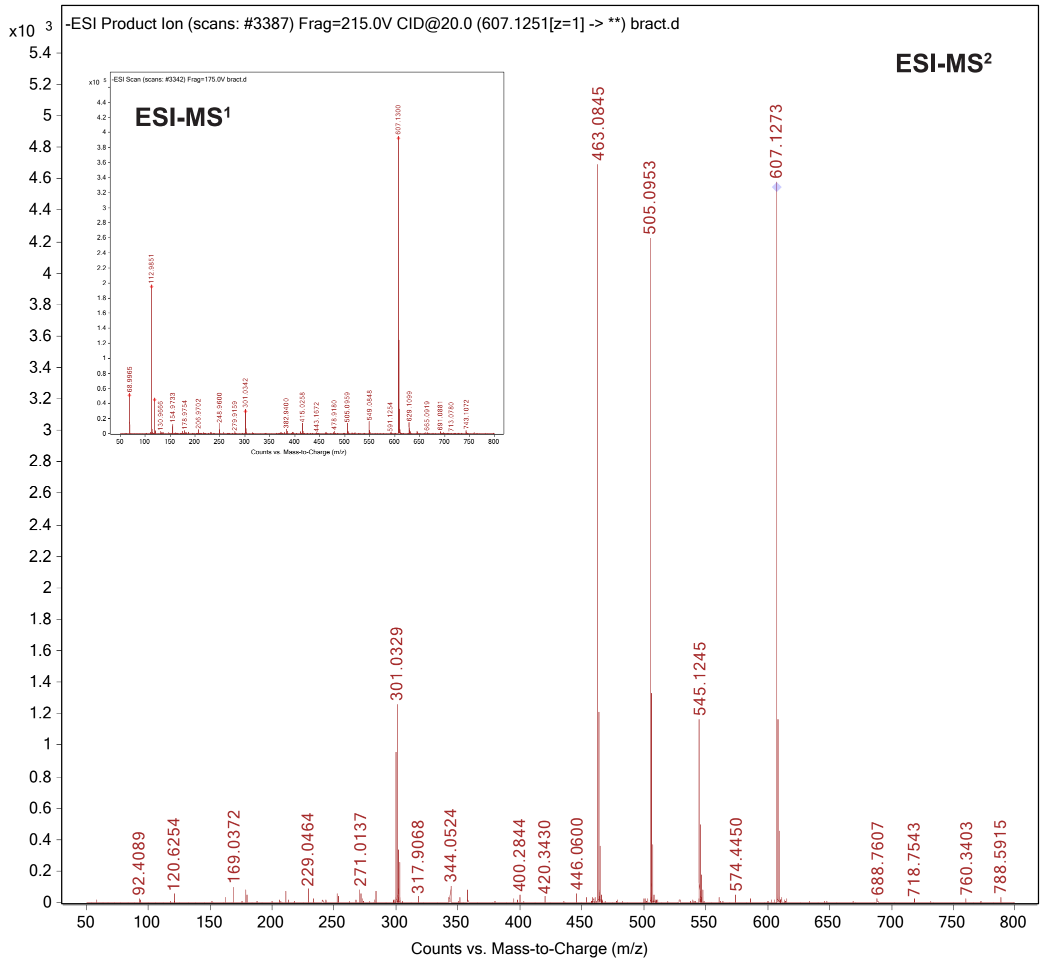

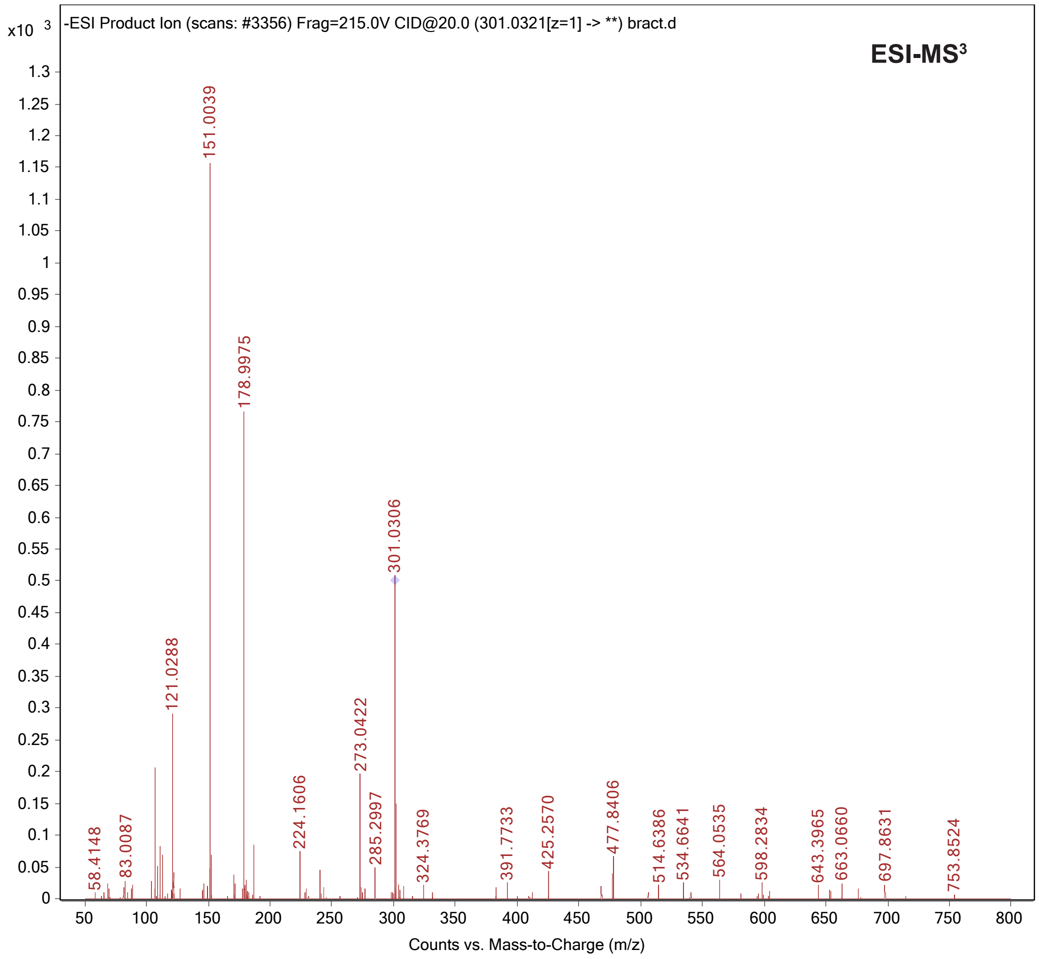

Supplementary Figure 10. The mass race (ES-) of LC-MS spectrum of compound 5 (Supplementary Figure 5) at 40, 20 and 10 eV.

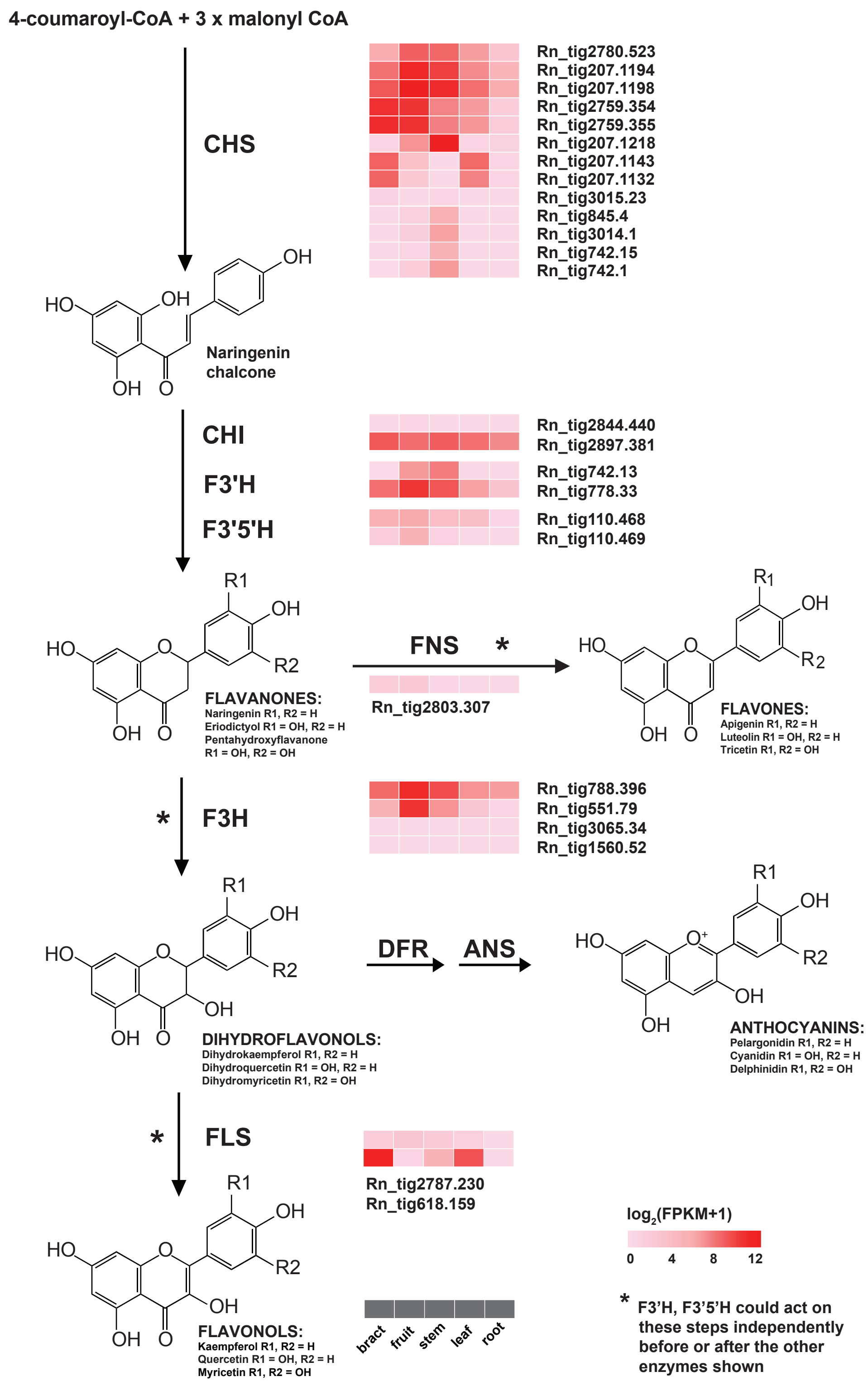

Supplementary Figure 11. Tissue specific expression of the flavonoid biosynthesis pathway in noble rhubarb. The flavo-noid biosynthesis pathway, and expression (log2(FPKM+1)) of genes in five tissues: bract, flower, stem, leaf and root. CHS: chalcone synthase, CHI: chalcone isomerase, F3'H: flavo-noid-3'-hydroxylase, F3H: flavanone-3-hydroxylase, FLS: flavonol synthase, F3'5'H: flavo-noid-3'5'-hydroxylase, FNS: flavone synthase, DFR: dihydroflavonol 4-reductase, and ANS: anthocyanidin synthase.

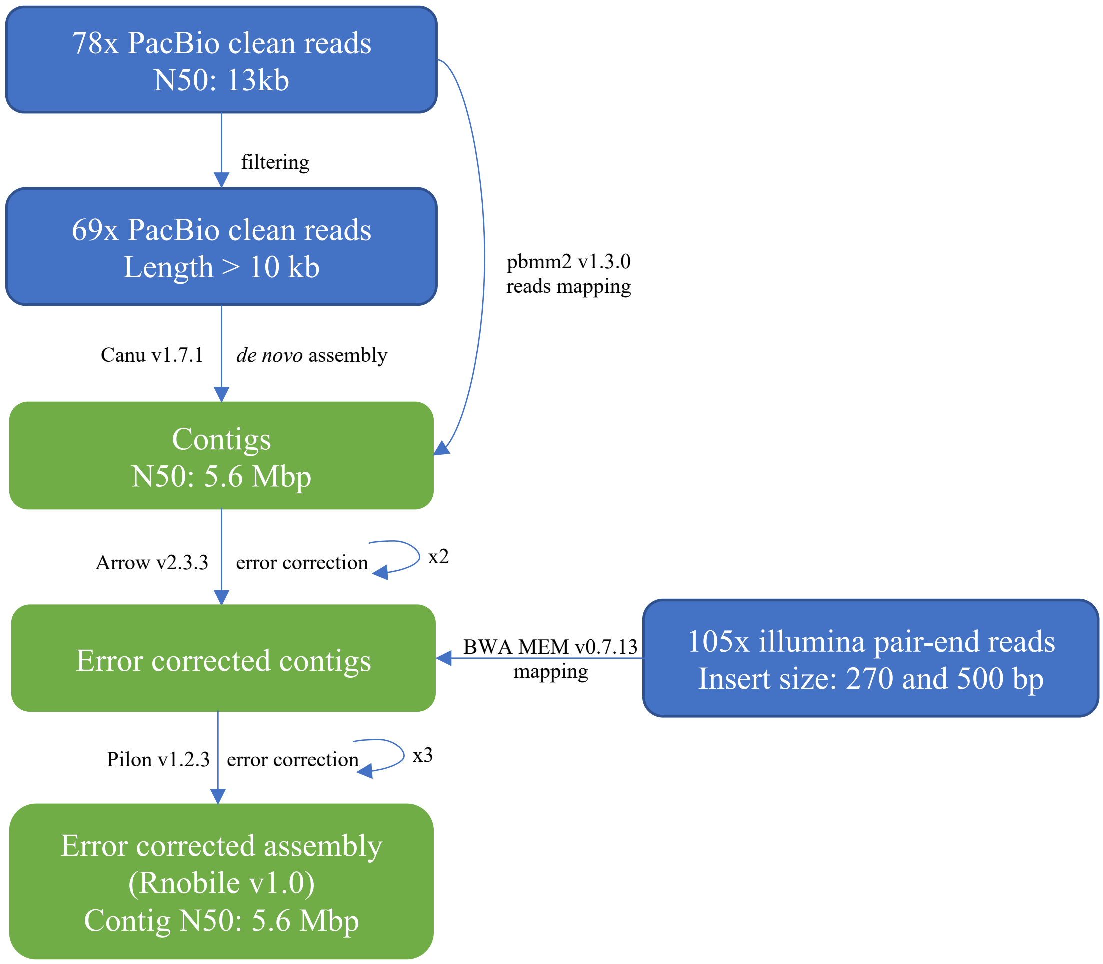

Supplementary Figure 12. Pipeline used to assemble the genome of noble rhubarb. The sequencing data used for assembly are shown in the blue boxes. Assembly results are shown in green boxes. Software and assem-bly processes are shown on the left and right side of each arrow, respectively.

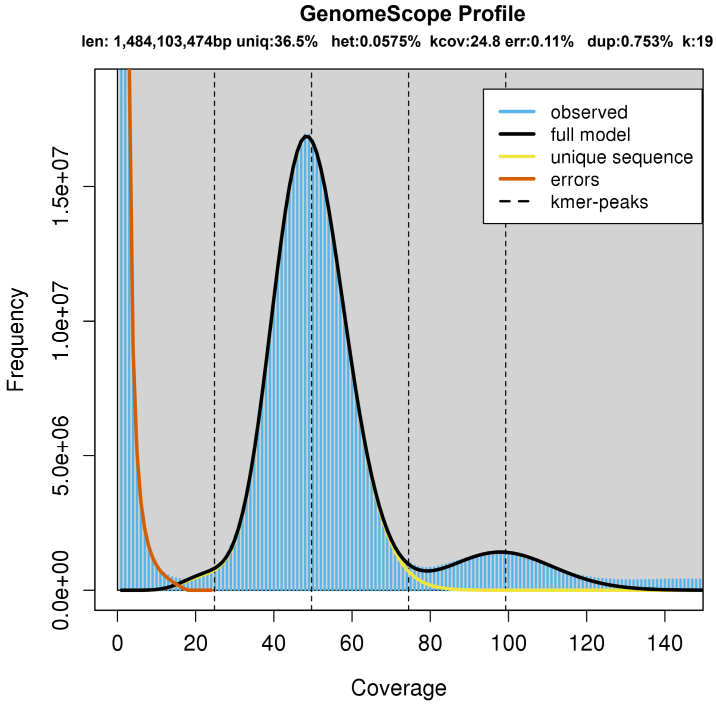

Supplementary Figure 13. Frequency distribution of k-mers (k = 19) using 86 Gbp high-quality Illumina paired-end reads.

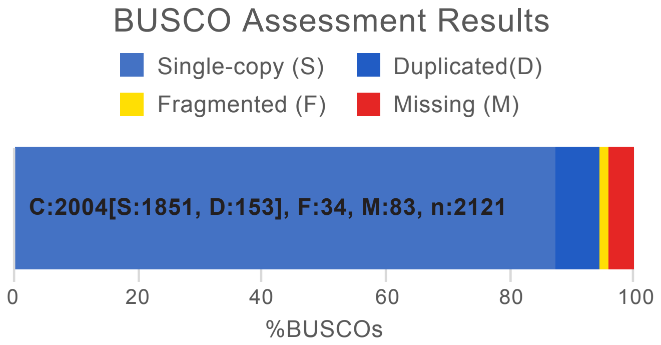

Supplementary Figure 14. BUSCO assessment result of the genome assembly of noble rhubarb.

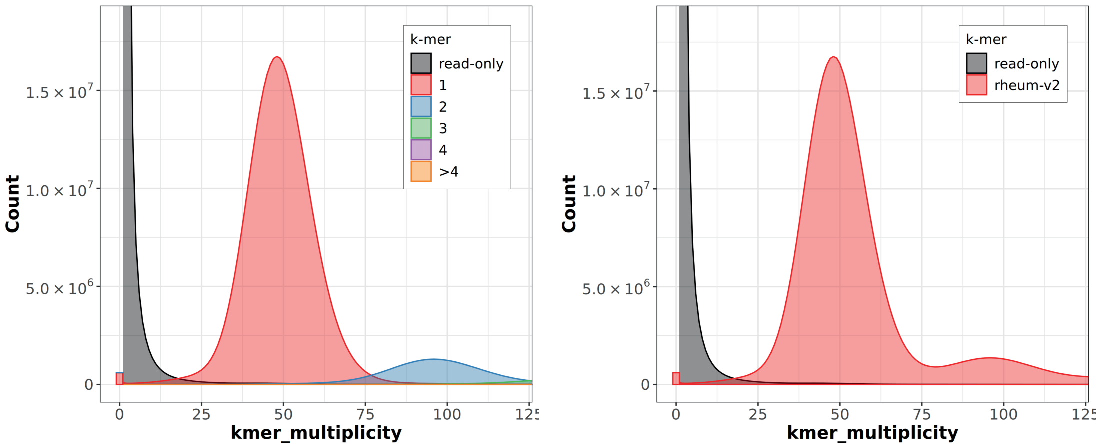

Supplementary Figure 15. Merqury spectra-cn (left) and spectra-asm (right) plot of the assembly. 48x sequencing cover-age (corresponding to the 1-copy peak) and 19-mer were used. The assembly k-mers absent from the read set (likely to be base errors in the assembly) are plotted as a bar at zero multiplici-ty, colored by the copy numbers found in the assembly.

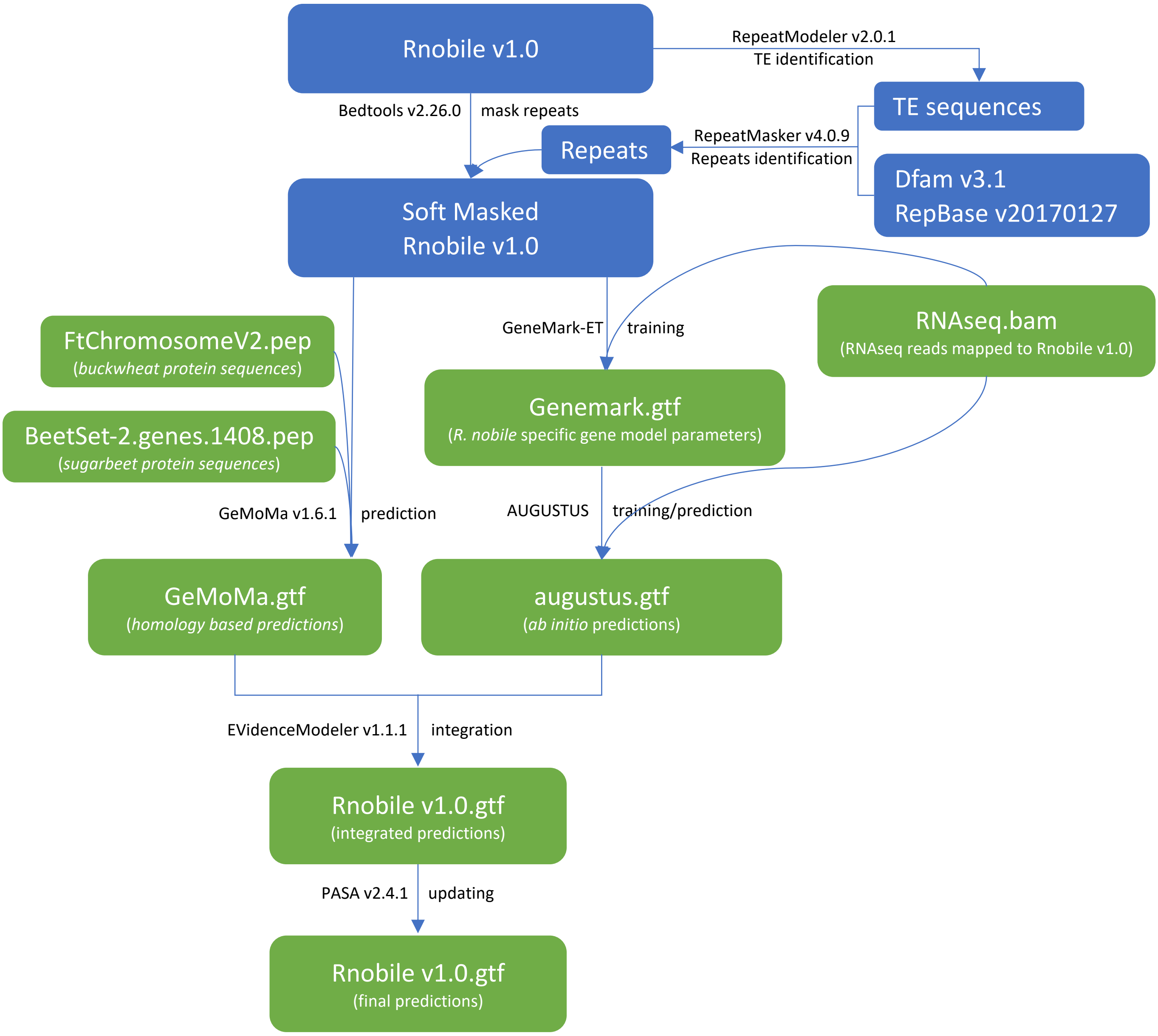

Supplementary Figure 16. Pipeline used to predict gene models from the genome assembly of noble rhubarb. The repeat identification processes are shown in the blue boxes. The prediction steps are shown in green boxes. Software and corresponding processes are shown on the left and right side of each ar-row, respectively.

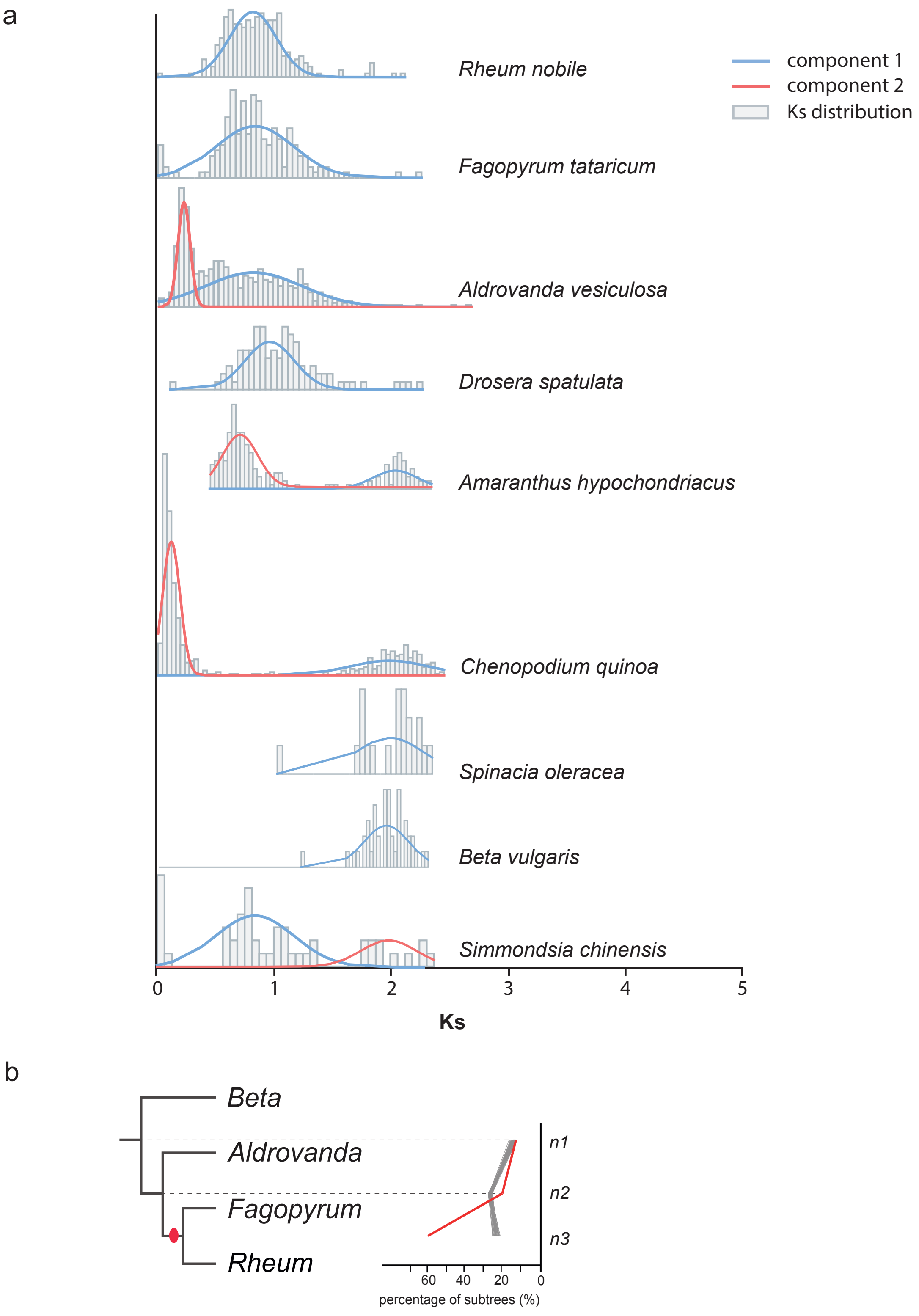

Supplementary Figure 17. Whole genome duplications in Caryophyllales species. **a.** Ks (synonymous substitution rates) plots of homologous colinear gene pairs within the genome sequences of Caryophyllales spe-cies.; **b.** MAPS results indicating percentage of subtrees that contain a gene duplication shared by descendant species at each node from observed data (red line), 100 resampled sets of null simulations (grey lines).

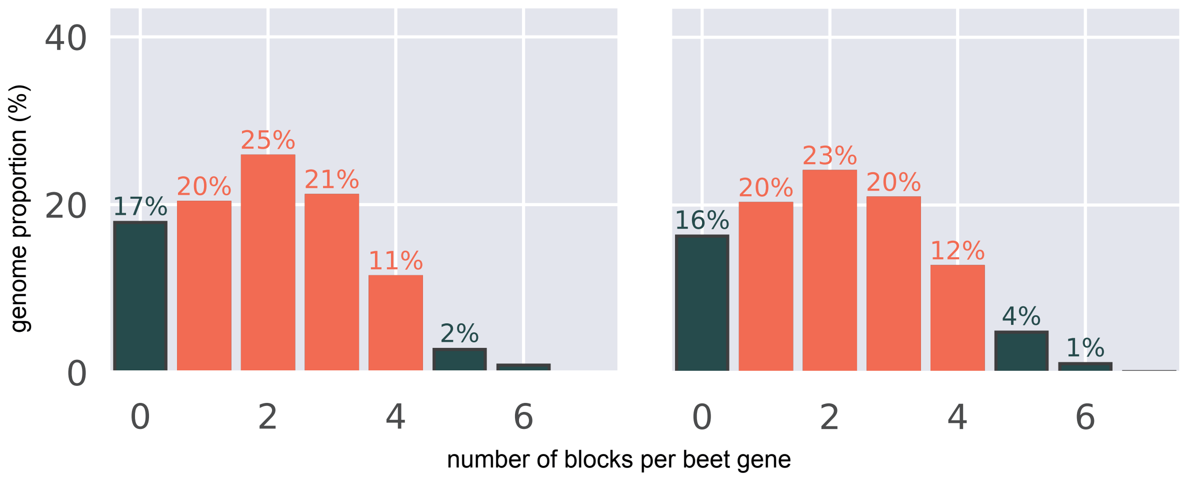

Supplementary Figure 18. The syntenic depth of collinear gene blocks between beet (*Beta vulgaris*) and *Fagopyrum tataricum* (left) or *Rheum nobile* (right).

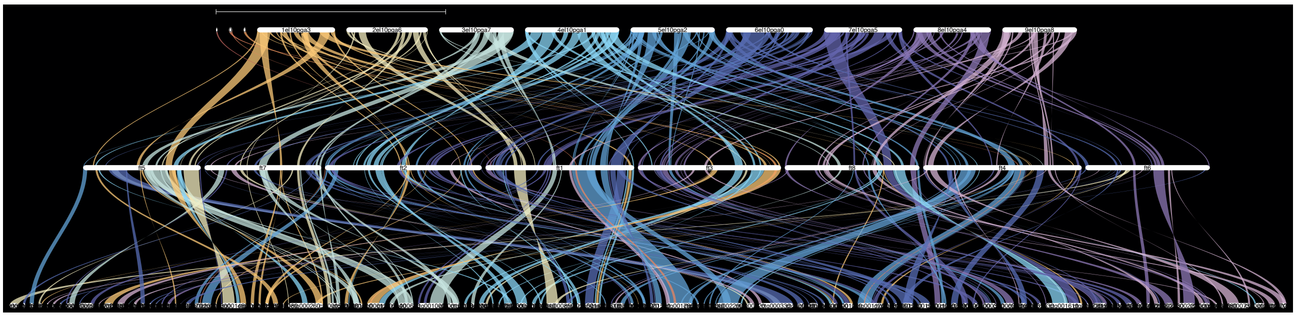

Supplementary Figure 19. Plot of overall genome synteny for *B. vulgaris* (top), *F. tataricum* (middle) and *R. nobile* (bottom).

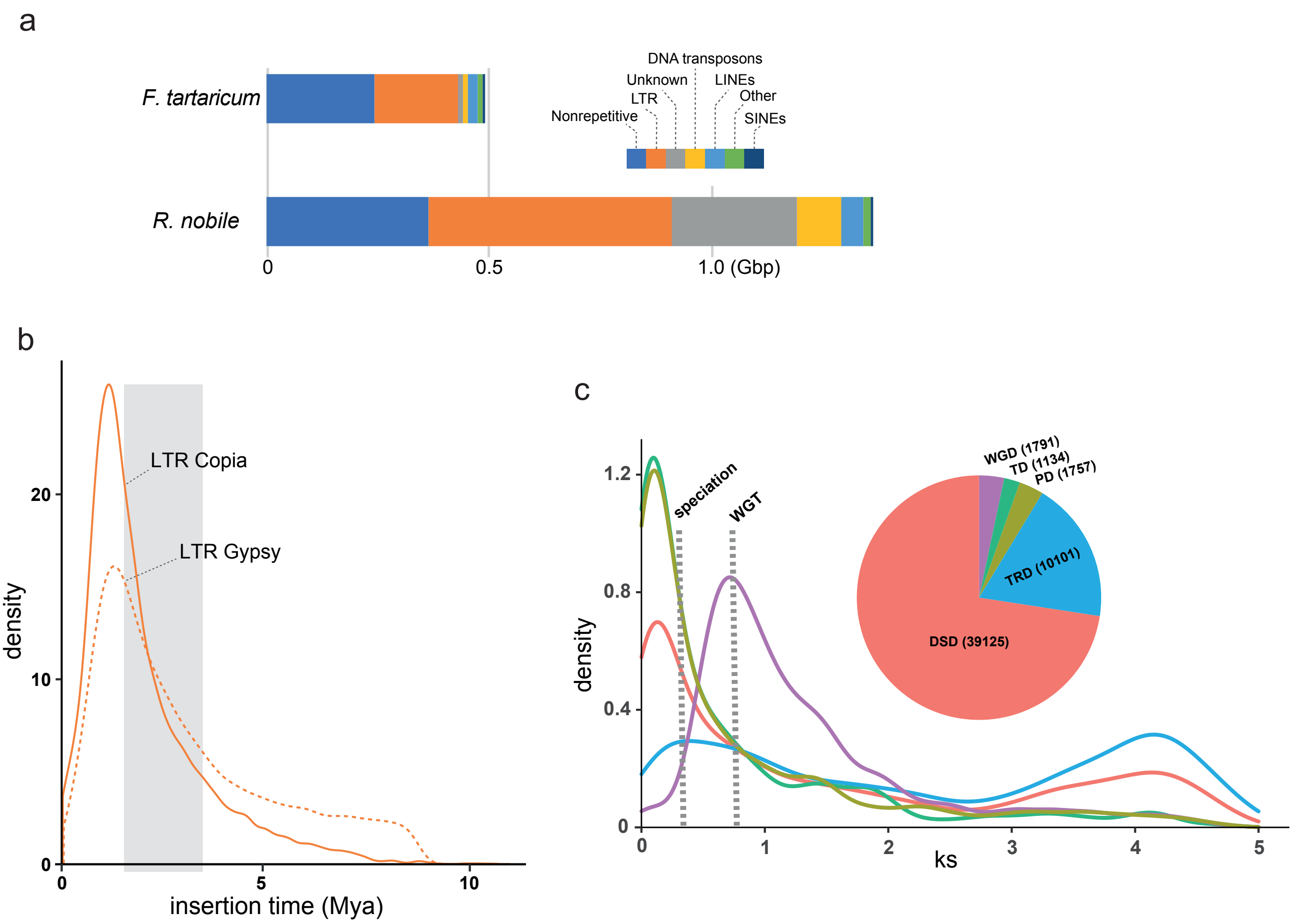

Supplementary Figure 20. Genome components of R. nobile. a. Genomic content (1C content in Gigabases) of repetitive versus nonrepetitive sequences in the *R. nobile* and *F. tataricum* genomes; b. The expansion history of LTR retrotransposons in the *R. nobile* genome. Grey shading indicates the time scale (1.6-3.6 Mya) when the most recent uplift of the Tibetan plateau occurred; c. The number of gene pairs derived from WGT vs. other modes of gene duplication in *R. nobile* genome, and Ks plot of the homologous gene pairs. TD: tandem duplication, DSD: dispersed duplication, TRD: transposed duplication, PD: proximal duplication, WGD: whole genome duplication.

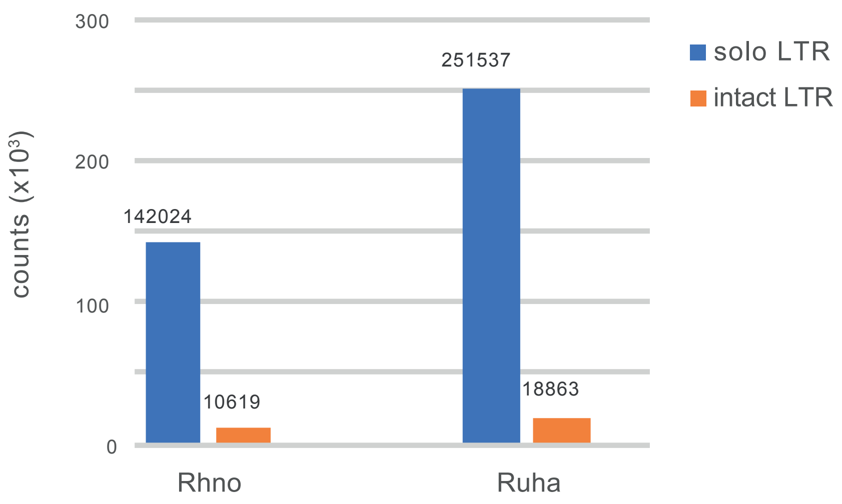

Supplementary Figure 21. Counts of LTR (solo and intact) identified in *R. nobile* (Rhno) and *Rumex hastatulus* (Ruha).

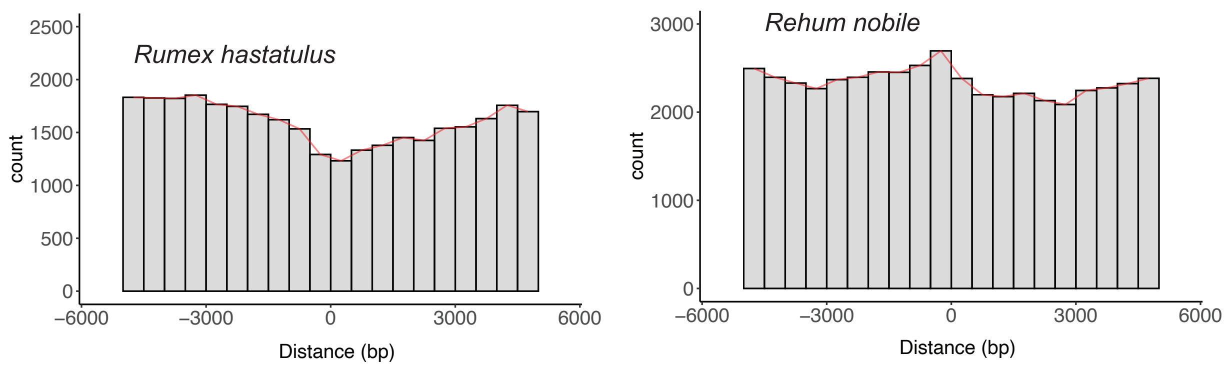

Supplementary Figure 22. Histogram plot of the counts of solo LTRs sorted by their distances to the near gene (bin width is 500 bp), showing that the insertion of solo LTRs were not significantly enriched in the flank-ing of gene.

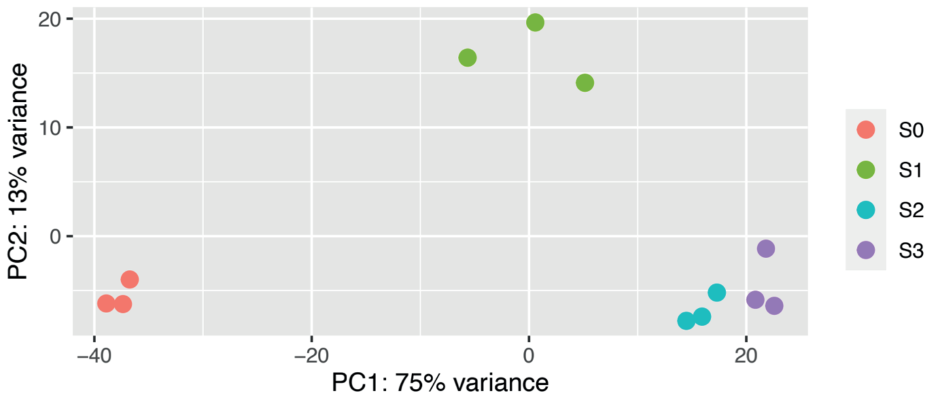

Supplementary Figure 23. PCA plot of RNA-seq profiles in four tissue sites of glasshouse morphology.
