# Supplementary material for: The genome of the glasshouse plant noble rhubarb (Rheum nobile) provides a window into alpine adaptation
Source: Commun Biol. 2023 Jul 10;6:706. doi: 10.1038/s42003-023-05044-1 (PMC10333194; doi:10.1038/s42003-023-05044-1)
Supplement: Supplementary file 2 — Description of Additional Supplementary Files [file 42003_2023_5044_MOESM2_ESM.pdf]

Description of Additional Supplementary Files

File name: Supplementary Data 1
Description: Species and genomes/transcriptomes used in the phylogenetic analysis.

File name: Supplementary Data 2
Description: Statistics for the Rheum nobile genome sequencing, assembly and annotation.

File name: Supplementary Data 3
Description: Genome data used in the comparative genomic analyses.

File name: Supplementary Data 4
Description: GO Enrichments of the significantly expanded gene families in R. nobile

File name: Supplementary Data 5
Description: GO Enrichments for the significantly down- and up-regulated genes in yellowish bracts compared to green leaves.

File name: Supplementary Data 6
Description: The source data behind the graphs in Figures 2-6.
