# Supplementary material for: The genome of the glasshouse plant noble rhubarb (Rheum nobile) provides a window into alpine adaptation
Source: Commun Biol. 2023 Jul 10;6:706. doi: 10.1038/s42003-023-05044-1 (PMC10333194; doi:10.1038/s42003-023-05044-1)
Supplement: Supplementary file 5 — Reporting Summary [file 42003_2023_5044_MOESM5_ESM.pdf]

nature portfolio

Corresponding author(s): Hengchang Wang
Last updated by author(s): Aug 16, 2022

Reporting Summary

Nature Portfolio wishes to improve the reproducibility of the work that we publish. This form provides structure for consistency and transparency in reporting. For further information on Nature Portfolio policies, see our Editorial Policies and the Editorial Policy Checklist.

Statistics

For all statistical analyses, confirm that the following items are present in the figure legend, table legend, main text, or Methods section.

n/a	Confirmed	
☐	☒	The exact sample size (*n*) for each experimental group/condition, given as a discrete number and unit of measurement
☐	☒	A statement on whether measurements were taken from distinct samples or whether the same sample was measured repeatedly
☐	☒	The statistical test(s) used AND whether they are one- or two-sided<br>*Only common tests should be described solely by name; describe more complex techniques in the Methods section.*
☒	☐	A description of all covariates tested
☒	☐	A description of any assumptions or corrections, such as tests of normality and adjustment for multiple comparisons
☐	☒	A full description of the statistical parameters including central tendency (e.g. means) or other basic estimates (e.g. regression coefficient) AND variation (e.g. standard deviation) or associated estimates of uncertainty (e.g. confidence intervals)
☐	☒	For null hypothesis testing, the test statistic (e.g. *F*, *t*, *r*) with confidence intervals, effect sizes, degrees of freedom and *P* value noted<br>*Give P values as exact values whenever suitable.*
☒	☐	For Bayesian analysis, information on the choice of priors and Markov chain Monte Carlo settings
☒	☐	For hierarchical and complex designs, identification of the appropriate level for tests and full reporting of outcomes
☒	☐	Estimates of effect sizes (e.g. Cohen's *d*, Pearson's *r*), indicating how they were calculated

Our web collection on statistics for biologists contains articles on many of the points above.

Software and code

Policy information about availability of computer code

Data collection	n/a
Data analysis	Genome raw reads clean: FastQC v0.11.8; Trimmomatic v0.39<br>Genome size estimation: Jellyfish v2.3.0; GenomeScope v1.0.0<br>Genome assembly, polishing, annotation and quality assessment: Canu v1.7.1; pbmm2 v1.3.0; GenomicConsensus v2.3.3; Pilon v1.23; RepeatModeler v2.0.1; RepeatMasker v4.0.9; QUAST v5.0.2; BUSCO v3.0.2; BRAKER2 pipeline; AUGUSTUS v3.2.1; GeMoMa v1.6.1; EVidenceModeler v1.1.1; PASA v2.4.1; InterProScan v5.40; Blast2Go v5.2; tRNAscan-SE v2.0.7; RNAmmer v1.2; INFERNAL v1.1.3<br>Transcritomics: FastQC v0.11.8; Trimmomatic v0.39; TransDecoder v5.5.0; CD-HIT-V4.6.1; HISAT2 v2.1.0; StringTie v2.0.5;<br>Comparative genomics: MCScanX; OrthoFinder v2.3.12; MAFFT v7.407; RAxML-NG v0.7.0b; CAFE v4.2.1; KIPEs pipeline v0.262; Trimal v1.2; FastTree v2.1.5

For manuscripts utilizing custom algorithms or software that are central to the research but not yet described in published literature, software must be made available to editors and reviewers. We strongly encourage code deposition in a community repository (e.g. GitHub). See the Nature Portfolio guidelines for submitting code & software for further information.

Data

Policy information about availability of data

All manuscripts must include a data availability statement. This statement should provide the following information, where applicable:

- Accession codes, unique identifiers, or web links for publicly available datasets
- A description of any restrictions on data availability
- For clinical datasets or third party data, please ensure that the statement adheres to our policy

Sequencing reads are available under the project CNP0001526 in China National GeneBank DataBase (CNGBdb) with accession number: CNR0350651-CNR0350660 (PacBio reads); CNR0350673-CNR0350676 (Illumina reads); CNR0350677-CNR0350686 (RNA-seq reads).

Human research participants

Policy information about studies involving human research participants and Sex and Gender in Research.

Reporting on sex and gender	n/a
Population characteristics	n/a
Recruitment	n/a
Ethics oversight	n/a

Note that full information on the approval of the study protocol must also be provided in the manuscript.

Field-specific reporting

Please select the one below that is the best fit for your research. If you are not sure, read the appropriate sections before making your selection.

☒ Life sciences ☐ Behavioural & social sciences ☐ Ecological, evolutionary & environmental sciences

For a reference copy of the document with all sections, see nature.com/documents/nr-reporting-summary-flat.pdf

Life sciences study design

All studies must disclose on these points even when the disclosure is negative.

Sample size	For genome sequencing and assembly of Rheum nobile, one individual is used to ensure the sample purity. This individual was also also used for transcriptome sequencing which were used in gene model prediction and annotation.
Data exclusions	Low quality genome sequencing reads were excluded for genome assembly following standard parctice.
Replication	For gene expression profiling of the glasshouse leaves, three replicates for each tissue sampling were used. In addition, we used three independent biological samples following standard practice in characterizing the glasshouse morphology.
Randomization	For the spectroscopic measurements and RNAseq, samples were assayed and processed randomly.
Blinding	Blinding was not deemed necessary for our study.

Reporting for specific materials, systems and methods

We require information from authors about some types of materials, experimental systems and methods used in many studies. Here, indicate whether each material, system or method listed is relevant to your study. If you are not sure if a list item applies to your research, read the appropriate section before selecting a response.

Materials & experimental systems

n/a	Involved in the study
☒	☐ Antibodies
☒	☐ Eukaryotic cell lines
☒	☐ Palaeontology and archaeology
☒	☐ Animals and other organisms
☒	☐ Clinical data
☒	☐ Dual use research of concern

Methods

n/a	Involved in the study
☒	☐ ChIP-seq
☒	☐ Flow cytometry
☒	☐ MRI-based neuroimaging
